# Prevalence and Genetic Basis of Antimicrobial Resistance in Non-*aureus* Staphylococci Isolated from Canadian Dairy Herds

**DOI:** 10.3389/fmicb.2018.00256

**Published:** 2018-02-16

**Authors:** Diego B. Nobrega, Sohail Naushad, S. Ali Naqvi, Larissa A. Z. Condas, Vineet Saini, John P. Kastelic, Christopher Luby, Jeroen De Buck, Herman W. Barkema

**Affiliations:** ^1^Department of Production Animal Health, Faculty of Veterinary Medicine, University of Calgary, Calgary, AB, Canada; ^2^Canadian Bovine Mastitis and Milk Quality Research Network, St-Hyacinthe, QC, Canada; ^3^Population, Public and Indigenous Health Strategic Clinical Network, Alberta Health Services, Calgary, AB, Canada; ^4^Department of Large Animal Clinical Sciences, Western College of Veterinary Medicine, University of Saskatchewan, Saskatoon, SK, Canada; ^5^Department of Reproduction, Obstetrics and Herd Health, Faculty of Veterinary Medicine, Ghent University, Ghent, Belgium

**Keywords:** antimicrobial resistance, antimicrobial resistance genes, coagulase-negative staphylococci, dairy, mastitis, non-*aureus* staphylococci, prevalence

## Abstract

Emergence and spread of antimicrobial resistance is a major concern for the dairy industry worldwide. Objectives were to determine: (1) phenotypic and genotypic prevalence of drug-specific resistance for 25 species of non-aureus staphylococci, and (2) associations between presence of resistance determinants and antimicrobial resistance. Broth micro-dilution was used to determine resistance profiles for 1,702 isolates from 89 dairy herds. Additionally, 405 isolates were sequenced to screen for resistance determinants. Antimicrobial resistance was clearly species-dependent. Resistance to quinupristin/dalfopristin was common in *Staphylococcus gallinarum* (prevalence of 98%), whereas *S. cohnii* and *S. arlettae* were frequently resistant to erythromycin (prevalence of 63 and 100%, respectively). Prevalence of resistance was 10% against β-lactams and tetracyclines. In contrast, resistance to antimicrobials critically important for human medicine, namely vancomycin, fluoroquinolones, linezolid and daptomycin, was uncommon (< 1%). Genes encoding multidrug-resistance efflux pumps and resistance-associated residues in deducted amino acid sequences of the *folP* gene were the most frequent mechanisms of resistance, regardless of species. The estimated prevalence of the *mecA* gene was 17% for *S. epidermidis*. Several genes, including *blaZ, mecA, fexA, erm, mphC, msrA*, and *tet* were associated with drug-specific resistance, whereas other elements were not. There were specific residues in *gyrB* for all isolates of species intrinsically resistant to novobiocin. This study provided consensus protein sequences of key elements previously associated with resistance for 25 species of non-*aureus* staphylococci from dairy cattle. These results will be important for evaluating effects of interventions in antimicrobial use in Canadian dairy herds.

## Introduction

Non-*aureus* staphylococci (NAS) have emerged as the most frequently isolated group of pathogens in intramammary infections (IMI) in dairy cows, with an estimated udder quarter-level prevalence of 26 cases per 100 quarters (Condas et al., 2017). Worldwide, mastitis remains one of the most frequent reasons for antimicrobial therapy in dairy herds (Mitchell et al., 1998; Brunton et al., 2012; Nobrega et al., 2017). Therefore, NAS are in relatively frequent contact with antimicrobials. Because of the high prevalence of these pathogens in the mammary gland, the presence of antimicrobial resistance (AMR) in this group of bacteria has potential to cause substantial damage to the dairy industry, animal health and welfare.

Whereas recent reports illustrate that NAS may harbor resistance determinants (Srednik et al., 2017b; Wipf et al., 2017) including genes considered potential public health hazards (Srednik et al., 2017a), the prevalence of AMR and respective resistance determinants in NAS isolated from dairy herds remains unknown. It is noteworthy that the vast majority of studies designed to estimate the prevalence of AMR in NAS isolated from dairy herds are regional and limit screening of resistance determinants to resistant isolates, usually by PCR or a similar approach. This methodology has two major limitations. First, PCR and its variants are usually designed to target few AMR genes (ARGs), limiting results to screened elements. Second, estimated prevalences of ARGs refer to the presence of genetic determinants in the phenotypically resistant population, whereas the same in the phenotypically susceptible population is unknown. The availability of such information would be important for interpreting patterns and trends of AMR, provide an estimate of the impact of various genes in drug-specific resistance, serve as a basis of risk assessment, and determine effects of interventions for controlling AMR.

Whether the presence of ARGs in NAS isolated from dairy cows poses an additional risk to human health is still unknown. An initial assessment relies on availability of information on AMR and resistance determinants in NAS isolated from dairy cattle. Data and samples collected from 89 dairy herds across Canada as part of a longitudinal study by the Canadian Bovine Mastitis Research Network provided a unique opportunity to study distribution of AMR, ARGs and other resistance determinants, e.g., mutations in NAS isolates. The objectives of this study were to: (1) estimate prevalence of drug-specific AMR in NAS isolated from Canadian dairy cows, (2) characterize genetic determinants of AMR associated with various drug-specific resistance profiles, and (3) study the association between the presence of resistance determinants and AMR in NAS isolated from Canadian dairy herds.

## Materials and methods

### Herds and sampling

Data for this study were obtained from the National Cohort of Dairy Farms of the Canadian Bovine Mastitis Research Network (Reyher et al., 2011). Eighty-nine herds from six Canadian provinces (Alberta, Ontario, Quebec, and the Maritimes provinces Prince Edward Island, New Brunswick and Nova Scotia) were selected to be representative of their respective province in terms of bulk tank somatic cell count, housing system, milking schedule, and breed of cattle. Herds were followed for almost 2 years from February 2007 to December 2008, and milk samples were collected following standardized procedures (Reyher et al., 2011; Saini et al., 2012b). Briefly, three sets of milk samples were collected. The first set included samples from all clinical mastitis cases observed for the duration of the study, as well as follow-up samples collected 2and 4 weeks after antimicrobial treatment. The second set included samples from 15 lactating cows per herd, systematically selected using a random sampling computer-driven method. Cows were re-sampled once every week (summer) or every 3 weeks (winter) for 6 weeks. The third set included samples from 15 cows sampled before drying off and after calving.

### Isolates and NAS identification

Overall, 115,294 milk samples were obtained longitudinally from 5,157 lactating cows. Bacteriological culture and identification were performed according to National Mastitis Council guidelines (National Mastitis Council, 1999). NAS were isolated from IMI (defined as ≥1,000 NAS cfu/mL of milk in pure culture) at least once from 2,091 cows. A random selection of one NAS isolate per cow was done, irrespective of the sampling set, resulting in 2,091 isolates obtained from 2,091 NAS-positive cows. From this total, 1,702 isolates were available to be included in the present study (57 from clinical and 1,645 non-clinical mastitis; including isolates from IMI and subclinical mastitis).

NAS isolates were previously characterized using partial sequencing of the *rpoB* gene and NCBI's BLAST, with a threshold of 97% identity was used to define a species (Condas et al., 2017). Where high identity for two distinct species was observed, full-length *rpoB* sequences were obtained and aligned against a database of 450 bovine NAS previously well-characterized (Naushad et al., 2016).

### Minimum inhibitory concentrations (MIC) determination

Phenotypic AMR was obtained using minimum inhibitory concentrations (MIC) for antimicrobials commonly administered to dairy cattle and humans, following the Clinical Laboratory and Standards Institute (CLSI) guidelines (Clinical Laboratory Standards Institute, 2016). Antimicrobials and concentrations evaluated were ampicillin (0.12–8 μg/ml), chloramphenicol (2–16 μg/ml), ceftiofur (0.5–4 μg/ml), cephalothin (2–16 μg/ml), ciprofloxacin (1–2 μg/ml), clindamycin (0.5–2 μg/ml), daptomycin (0.5–4 μg/ml), erythromycin (0.25–4 μg/ml), gentamicin (2–16 μg/ml), levofloxacin (0.25–4 μg/ml), linezolid (1–8 μg/ml), moxifloxacin (0.25–4 μg/ml), nitrofurantoin (32–64 μg/ml), oxacillin + 2% NaCl (0.25–4 μg/ml), penicillin (0.06–8 μg/ml), penicillin/novobiocin (1/2–8/16 μg/ml), pirlimycin (0.5–4 μg/ml), quinupristin/dalfopristin (0.5–4 μg/ml), rifampin (0.5–4 μg/ml), tetracycline (2–16 μg/ml), tigecycline (0.03–0.5 μg/ml), trimethoprim/sulfamethoxazole (0.05/9.5–4/76 μg/ml), and vancomycin (0.25–32 μg/ml). Positive controls (antimicrobial-free wells) were included in all plates. *Staphylococcus aureus* ATCC 29213 was used as quality control strain. Breakpoints were defined according to CLSI (Clinical Laboratory Standards Institute, 2008, 2016) with slight modifications (*S. aureus* oxacillin breakpoint was used instead of the coagulase-negative staphylococci breakpoint). For antimicrobials not described by CLSI (i.e., tigecycline), European Committee on Antimicrobial Susceptibility Testing (EUCAST) breakpoints were used (European Committee on Antimicrobial Susceptibility Testing, 2017). AMR was defined as non-susceptibility to a given antimicrobial by combining intermediate and resistant categories into a single category. Therefore, isolates with MIC values equal or above the intermediate breakpoint were classified as resistant (Table S1). Multidrug resistance (MDR) was defined as resistance to at least three distinct antimicrobial classes.

### Presence of antimicrobial resistance determinants

**Figure 1:**
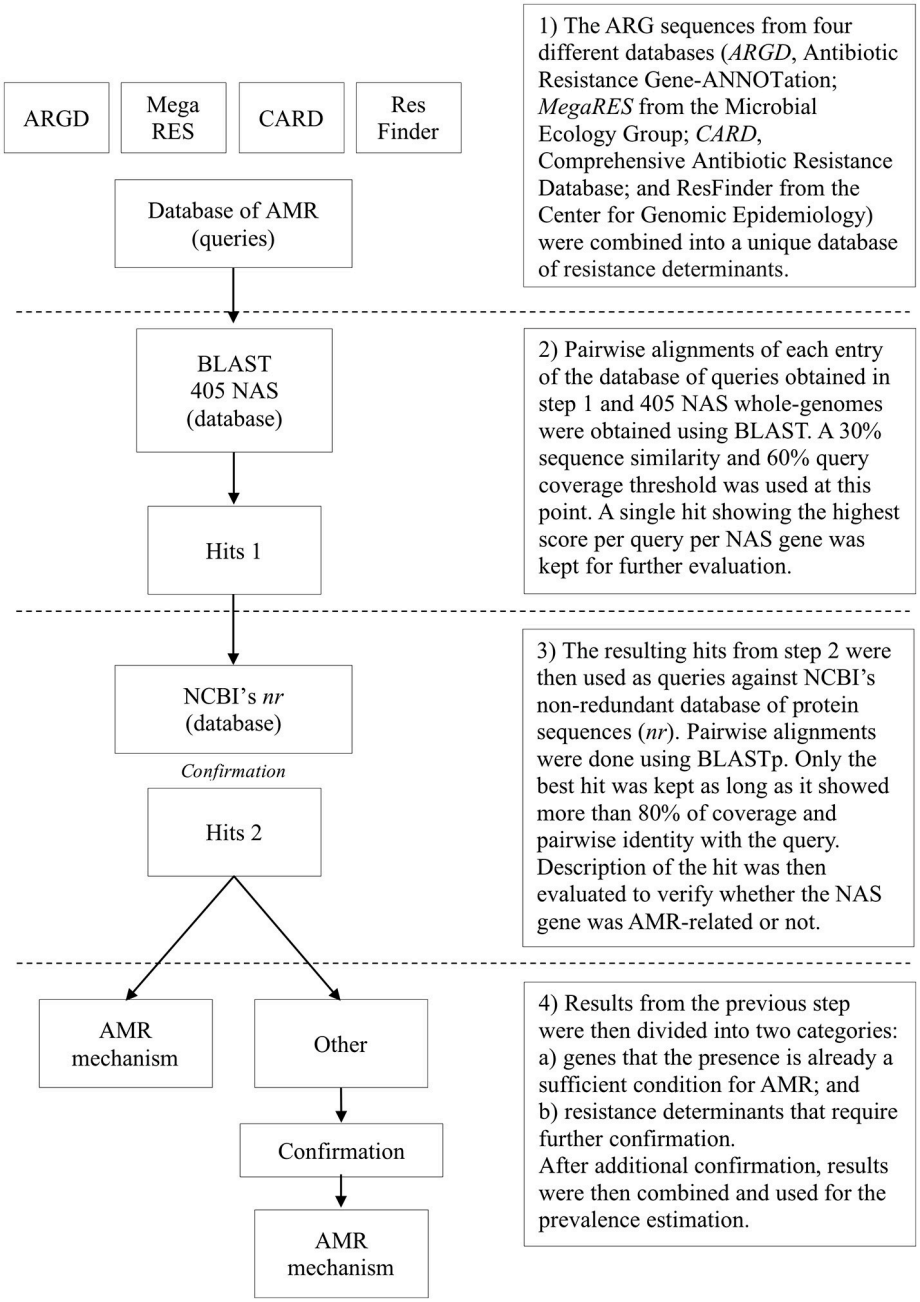
Flow diagram for determining genetic elements associated with antimicrobial resistance (AMR) in 405 bovine non-*aureus* staphylococci (NAS).

Overall, 405 NAS (348 non-clinical mastitis and all 57 clinical mastitis isolates) were sequenced using a MiSeq platform after sample preparation using the Nextera XT DNA Library Prep kit from Illumina®. Selection was based on the inclusion of all clinical mastitis isolates available (*n* = 57), inclusion of all isolates for uncommon species (< 20 unique isolates available at the cow-level), random selection of one isolate for all other species until 385 isolates were included, and a final random selection of 20 MDR isolates. Genomes were assembled following a standardized protocol and submitted to NCBI under BioProject ID PRJNA342349 (Naushad et al., 2016). Prevalence of ARGs and other resistance determinants was evaluated using data from 4 databases: (1) ARG-ANNOT v3 (Antibiotic Resistance Gene-ANNOTation) (Gupta et al., 2014); (2) MegaRES v1.0.1 (Lakin et al., 2017); (3) Comprehensive Antibiotic Resistance Database v1.1.6 (CARD) (Jia et al., 2017); and (4) ResFinder from the Center for Genomic Epidemiology (as of November 02, 2016) (Zankari et al., 2012). These four databases were merged into a single database containing ARGs protein sequences. Each entry was used as a query against the 405 NAS genomes using a personalized BLAST server. Initially, a single best hit for each query for each NAS genome was retrieved using a 30% similarity and 60% query coverage threshold. To prevent two similar but distinct queries returning hits to the same gene within a particular genome, each region of the genome could only map to a single query. Following this logic, if two queries returned strong hits for the same gene within the same isolate, one was discarded after comparison of the hits results. Hits were compared using a score defined as the product between pairwise identity and query coverage, and the hit with the highest score was selected. Next, the protein sequences of all returned hits were used as queries against the non-redundant database (*nr*) using NCBI's BLASTp (Altschul et al., 1990), where the best hit was considered definitive, provided it had >80% coverage and percent identity with the query. For resistance determinants that required additional confirmation (substitutions, residues composition), pairwise alignments were done using MEGA 7.0 (Kumar et al., 2016) where the presence of specific residues associated with AMR in *Staphylococcus* spp. was confirmed against reference sequences available in literature (Hampele et al., 1997; Aubry-Damon et al., 1998; Li et al., 1998; Schmitz et al., 1998; Guirao et al., 2001; Linde et al., 2001; Roychoudhury et al., 2001; Fujimoto-Nakamura et al., 2005; Trong et al., 2005; Friedman et al., 2006; Mwangi et al., 2007; Vickers et al., 2007; Yamada et al., 2008; Peleg et al., 2012; Zhou et al., 2012; Davlieva et al., 2013; Kwak et al., 2013) (Figure 1). The following criteria were used to associate the presence of known mutations with AMR: (1) residues at a specific position previously associated with AMR in coagulase-negative staphylococci (CNS); and (2) residues at a specific position previously associated with AMR in any *Staphylococcus* spp. In order to screen for novel residues associated with AMR, protein sequences from resistant and susceptible isolates of the same species were aligned and the presence of residues were described for both groups.

### Statistical analyses

All statistical analyses were done using R 3.4.1 (R Core Team, 2017) and Stata 15 (Statacorp, 2017). Packages used for analyses included *lme4* (Bates et al., 2015) and *geepack* (Halekoh et al., 2006). The raw data supporting the conclusions of this manuscript will be made available upon request.

#### Prevalence estimation of phenotypic and genotypic AMR

Prevalence or proportion of positive isolates was obtained using statistical models at the isolate level and expressed in percentage points. For prevalence estimation of ARGs and other resistance determinants, MDR isolates that were selected purposely were initially excluded, resulting in 385 NAS isolates (328 and 57 isolated from non-clinical and clinical mastitis, respectively). Generalized linear mixed models with herd-specific random effects were used to deal with the hypothetical lack of independence of isolates from the same herd. Models were fit separately for each species and antimicrobial (or resistance determinant) combination via maximum likelihood, with 30 quadrature points per scalar using the adaptive Gauss-Hermite quadrature (Bates et al., 2015). Estimated logits as well as its respective 95% confidence interval were converted back to proportions for presentation. For non-varying (all positive or all negative), and rare or uncommon outcomes (< 6 positive results), prevalence and its respective 95% exact confidence interval were estimated using the binomial distribution, where observations from different cows in the same herd were assumed to be independent. An initial assessment was done to compare results from clinical and non-clinical samples. Conditional on the species, no significant differences in the prevalence of drug-specific AMR or resistance determinants were observed when contrasting the two types of samples, non-clinical and clinical mastitis samples. Hence, they were combined for further analyses. As the residue composition of the AMR proteins is species-specific for NAS (Linde et al., 2001), the prevalence of mutations was only calculated for residues previously described as associated with AMR in *Staphylococcus* spp.

Finally, for prevalence estimation of the resistance determinants for the combined NAS, design weights were used at the isolate level to properly adjust for the possible bias introduced by the selection protocol, which favored the inclusion of a wide range of species. Weights were obtained using the ratio between the expected and observed proportion of a species being included in the sequencing protocol. The expected distribution of species was estimated from cow-level prevalence previously reported (Condas et al., 2017). Final weights were scaled to the sum of the total number of isolates sequenced in a particular herd (Carle, 2009). Weights were then used as scaled probability weights at the lowest level, using the *melogit* function in Stata 15.

#### Association between phenotypic AMR and genetic determinants of resistance

Marginal models using generalized estimating equations (GEE) were used to estimate the difference in the logit of being resistant between isolates that did and did not harbor a resistance determinant. Resistance was used as a binary variable, whereas the variance function was defined according to the Bernoulli distribution. The logit link was used for mean modeling, and the log(OR) was used in an exchangeable structure of the covariance-matrix to account for the lack of independence introduced by isolates from the same herd (Lipsitz et al., 1991). Sandwich estimators of the variances were used to deal with any misspecification of covariance structures. Scaled probability weights used for prevalence estimation were used to adjust for the possible bias introduced by the selection protocol of sequenced isolates. Drug-specific AMR was considered the outcome, whereas the resistance determinants associated with the respective resistance profile were considered single predictors in separate analyses (i.e., *tetK* as the single predictor and tetracycline resistance as the outcome). For drug-specific AMR with < 6 positive outcomes or in the presence of zero cells, Fisher's Exact test was used.

To compare the estimated difference in the logit of being resistant conditional on the presence of >1 genetic mechanism, only resistance determinants associated with AMR at *P* < 0.10 were considered. Initially, pattern tables were generated where possible combinations between candidate genes were first observed. Generalized estimating equations were used as described above, with drug-specific AMR as an outcome. Genes as well as observed interactions were considered predictors. For these analyses, there was an implicit assumption that observed effects were neither confounded nor modified by resistance determinants not considered in the model. For all analyses, *P* < 0.05 was considered significant and *P*-values were adjusted for multiple comparisons, as described (Benjamini and Hochberg, 1995).

## Results

### Prevalence of phenotypic AMR

On average, 19 NAS isolates per herd were included, ranging from four to 41 NAS per herd. The prevalence of AMR was highest for tetracycline, penicillin (10% for each), and erythromycin (6%) (Table 1). *Staphylococcus arlettae* had the highest prevalence of AMR, particularly against penicillin (61%), ampicillin (23%), erythromycin (100%), pirlimycin (18%) and clindamycin (99.9%). Prevalence of quinupristin/dalfopristin resistance was low or absent for all species except for *S. gallinarum* (98%) and *S. sciuri* (19%). Prevalence of MDR in NAS was 2 per 100 isolates, with *S. arlettae* (61%), *S. epidermidis* (6%), and *S. saprophyticus* (6%) being the most resistant species of the NAS commonly isolated from Canadian dairy herds (Table 1). No isolate was resistant to gentamicin, linezolid, cephalothin, vancomycin, levofloxacin, moxifloxacin, ciprofloxacin, or nitrofurantoin. Five (10%) *S. sciuri* isolates were resistant to daptomycin, whereas two *S. simulans* and one *S. epidermidis* were tigecycline-resistant. Five (0.3%) NAS isolates were resistant to the combination of trimethoprim and sulfamethoxazole (three *S. epidermidis* and two *S. vitulinus*). A single *S. haemolyticus* isolate was resistant to rifampin. Three *S. epidermidis*, one *S. chromogenes* and one *S. saprophyticus* were ceftiofur-resistant, and one of the three *S. epidermidis* isolates was also resistant to oxacillin. MIC_50_, MIC_90_ as well as range of drug-specific MIC values for the most common NAS species are presented in Table S2.

**Table 1:** Prevalence (Prev) of the phenotypic antimicrobial resistance per isolate for 25 non-*aureus* staphylococci species isolated from bovine quarter milk samples collected in 89 Canadian dairy herds.

**Species**	***N***	**CHL^a^**		**TET**		**Q-D**		**CLI**		**PNV**	
		**Prev**	**95% CI**	**Prev**	**95% CI**	**Prev**	**95% CI**	**Prev**	**95% CI**	**Prev**	**95% CI**
*S. chromogenes*	774	0.05	0.03–0.07	0.02	0.01–0.04	0	0–0.01	0.01	0–0.03	0.02	0.01–0.04
*S. simulans*	216	0.06	0.02–0.14	0.13	0.08–0.21	0	0–0.03	0	0–0.03	0	0–0.02
*S. xylosus*	209	0.01	0–0.04	0.31	0.22–0.41	0.04	0.01–0.11	0.04	0.01–0.12	0.01	0–0.03
*S. haemolyticus*	153	0.01	0–0.04	0.05	0.02–0.09	0.01	0–0.04	0.02	0–0.06	0.01	0–0.04
*S. epidermidis*	65	0.03	0–0.11	0.32	0.20–0.47	0.02	0–0.08	0.08	0.03–0.17	0.03	0–0.11
*S. cohnii*	61	0.05	0.01–0.14	0.16	0.05–0.41	0.08	0.03–0.18	0.07	0.02–0.16	0	0–0.06
*S. sciuri*	51	0.02	0–0.10	0.10	0.03–0.21	0.19	0.09–0.36	0.02	0–0.90	0	0–0.07
*S. capitis*	20	0	0–0.17	0.05	0–0.25	0	0–0.17	0	0–0.17	0	0–0.17
*S. gallinarum*	20	0.10	0.01–0.32	0.10	0.01–0.32	0.98	0.04–1.00	0	0–0.17	0	0–0.17
*S. warneri*	19	0	0–0.18	0.05	0–0.26	0	0–0.18	0.11	0.01–0.33	0	0–0.18
*S. saprophyticus*	18	0	0–0.19	0.39	0.20–0.62	0	0–0.19	0	0–0.19	0	0–0.19
*S. arlettae*	17	0.06	0–0.29	0.24	0.07–0.50	0	0–0.20	0.99	0–1.00	0	0–0.20
*S. succinus*	13	0	0–0.25	0	0–0.25	0	0–0.25	0	0–0.25	0	0–0.25
*S. agnetis*	11	0.09	0–0.41	0	0–0.28	0	0–0.28	0	0–0.28	0	0–0.28
*S. hominis*	11	0	0–0.28	0.36	0.11–0.69	0	0–0.28	0	0–0.28	0	0–0.28
*S. devriesei*	9	0	0–0.34	0.22	0.03–0.60	0	0–0.34	0	0–0.34	0	0–0.34
*S. equorum*	8	0	0–0.37	0.25	0.03–0.65	0	0–0.37	0	0–0.37	0	0–0.37
*S. vitulinus*	7	0	0–0.41	0	0–0.41	0	0–0.41	0	0–0.41	0	0–0.41
*S. pasteuri*	6	0	0–0.46	0.50	0.12–0.88	0	0–0.46	0	0–0.46	0	0–0.46
*S. hyicus*	4	0	0–0.60	0.25	0.01–0.81	0	0–0.60	0	0–0.60	0	0–0.60
*S. auricularis*	3	0	0–0.71	0	0–0.71	0	0–0.71	0	0–0.71	0	0–0.71
*S. nepalensis*	3	0	0–0.71	0.67	0.09–0.99	0	0–0.71	0.67	0.09–0.99	0	0–0.71
*S. caprae*	2	0	0–0.84	0	0–0.84	0	0–0.84	0	0–0.84	0	0–0.84
*S. fleuretti*	1	0	0–0.98	0	0–0.98	0	0–0.98	0	0–0.98	0	0–0.98
*S. kloosii*	1	0	0–0.98	0	0–0.98	0	0–0.98	0	0–0.98	0	0–0.98
Total	1,702	0.04	0.03–0.05	0.10	0.08–0.12	0.02	0.01–0.03	0.03	0.02–0.04	0.01	0–0.02
**Species**	***N***	**PIR^a^**		**ERY**		**AMP**		**PEN**		**MDR**	
		**Prev**	**95% CI**	**Prev**	**95% CI**	**Prev**	**95% CI**	**Prev**	**95% CI**	**Prev**	**95% CI**
*S. chromogenes*	774	0.01	0–0.03	0.01	0–0.03	0.09	0.07–0.13	0.13	0.1–0.17	0	0–0.02
*S. simulans*	216	0.01	0–0.09	0.02	0.01–0.05	0	0–0.03	0.01	0–0.03	0.01	0–0.04
*S. xylosus*	209	0.11	0.05–0.20	0.16	0.10–0.25	0.02	0.01–0.05	0.02	0.01–0.05	0.03	0–0.10
*S. haemolyticus*	153	0.03	0.01–0.07	0.02	0–0.06	0.03	0.01–0.07	0.14	0.07–0.26	0.01	0–0.05
*S. epidermidis*	65	0.01	0–0.60	0	0–0.59	0.08	0.03–0.17	0.22	0.13–0.33	0.06	0.02–0.15
*S. cohnii*	61	0.01	0–0.77	0.63	0.42–0.80	0	0–0.06	0.13	0.04–0.36	0.01	0–0.59
*S. sciuri*	51	0.13	0.04–0.34	0.04	0–0.13	0	0–0.07	0.02	0–0.10	0.02	0–0.10
*S. capitis*	20	0.05	0–0.25	0	0–0.17	0	0–0.17	0	0–0.17	0	0–0.17
*S. gallinarum*	20	0	0–0.17	0.05	0–0.25	0	0–0.17	0.05	0–0.25	0	0–0.17
*S. warneri*	19	0.05	0–0.26	0.11	0.01–0.33	0.11	0.01–0.33	0.11	0.01–0.33	0.05	0–0.26
*S. saprophyticus*	18	0.17	0.04–0.41	0	0–0.19	0	0–0.19	0.22	0.06–0.48	0.06	0–0.27
*S. arlettae*	17	0.18	0.04–0.43	1.00	0.80–1.00	0.23	0.07–0.50	0.61	0.25–0.88	0.61	0.25–0.88
*S. succinus*	13	0	0–0.25	0	0–0.25	0	0–0.25	0	0–0.25	0	0–0.25
*S. agnetis*	11	0	0–0.28	0	0–0.28	0	0–0.28	0	0–0.28	0	0–0.28
*S. hominis*	11	0	0–0.28	0	0–0.28	0	0–0.28	0	0–0.28	0	0–0.28
*S. devriesei*	9	0.22	0.03–0.60	0	0–0.34	0	0–0.34	0	0–0.34	0	0–0.34
*S. equorum*	8	0	0–0.37	0.87	0.46–0.98	0	0–0.37	0	0–0.37	0	0–0.37
*S. vitulinus*	7	0	0–0.41	0	0–0.41	0	0–0.41	0	0–0.41	0	0–0.41
*S. pasteuri*	6	0.17	0–0.64	0	0–0.46	0	0–0.46	0.17	0–0.64	0	0–0.46
*S. hyicus*	4	0	0–0.60	0	0–0.60	0	0–0.60	0	0–0.60	0	0–0.60
*S. auricularis*	3	0.33	0.01–0.91	0	0–0.71	0	0–0.71	0.33	0.01–0.91	0	0–0.71
*S. nepalensis*	3	0	0–0.71	0.67	0.09–0.99	0	0–0.71	0	0–0.71	0.33	0.01–0.91
*S. caprae*	2	0	0–0.84	0	0–0.84	0	0–0.84	0	0–0.84	0	0–0.84
*S. fleuretti*	1	0	0–0.98	0	0–0.98	0	0–0.98	0	0–0.98	0	0–0.98
*S. kloosii*	1	0	0–0.98	0	0–0.98	0	0–0.98	0	0–0.98	0	0–0.98
Total	1,702	0.03	0.02–0.05	0.06	0.05–0.08	0.05	0.04–0.07	0.10	0.09–0.13	0.02	0.01–0.03

a *CHL, Chloramphenicol; TET, tetracycline; Q-D, quinupristin/dalfopristin combination; CLI, clindamycin; PNV, penicillin/novobiocin combination; PIR, pirlimycin; ERY, erythromycin; AMP, ampicillin; PEN, penicillin; MDR, multidrug resistance*.

### Overall frequency of resistance determinants

The most common genetic basis of resistance included the presence of AMR-associated residues in the dihydropteroate synthase gene deduced amino acid sequence (*folP* gene; all sequenced isolates, ranging from two to six residues), the putative multidrug export ATP-binding/permease protein SAV1866 (99% of the isolates), the major facilitator superfamily (MFS) multidrug efflux transporter NorA represented by the *norA* gene (91% of the isolates) and the DHA sub-family of MFS transporters (61% of the isolates). Drug-specific efflux pumps-coding genes identified included *tet38, tetK*, and *tetL* (21, 12, and 3% of all NAS sequenced, respectively), the *mrsA* gene (42 isolates; 10%), and the chloramphenicol/florfenicol efflux MFS transporter FexA represented by the *fexA* gene (five isolates; 1%). Non-synonymous mutations in the quinolone resistance-determining region (QRDR) previously reported as associated with AMR were present for the *parC* and *parE* genes in isolates of *S. devriesei* and *S. epidermidis*, respectively. *gyrB* residues associated with resistance against aminocoumarins were present as the dominant pattern for several species intrinsically resistant to novobiocin. No AMR-associated residue was detected in the deduced amino acid sequence of the *rpoB, rpoC* and *gyrA* genes. *erm* genes, which encode for rRNA adenine N-6-methyltransferases, were present exclusively in *S. epidermidis, S. cohnii, S. equorum*, and *S. chromogenes*. No MLS-resistance mechanisms were present in *S. gallinarum*. The ABC-transporter encoding *vgaA* was detected in six isolates, whereas the virginiamycin B lyase encoding *vgbB* was present in a single *S. xylosus* isolate. *van* elements associated with vancomycin-resistance were not detected. The *mecA1* gene was present in all *S. sciuri* isolates and the *mecA sf* was present in all *S. fleuretti*. No mutation was detected in the promoter region of these genes.

### Prevalence of resistance determinants

The most prevalent resistance determinants, other than MDR efflux pumps and any AMR-associated residues, were *tet38* efflux pumps (30%), macrolide phosphotransferase C (*mphC*; 10%) and the β-lactamase gene *blaZ* (6%) (Table 2). The prevalence of these elements varied systematically according to species (Table S3). The *mecA* gene was present in four *S. epidermidis*, with an estimated prevalence of 17% for that species. Aminoglycoside-inactivating enzymes had an estimated prevalence close to zero, although they were present in all isolates for some species (*aph(3*′*)* gene for *S. capitis*; Table S3). The *mphC* and *msrA* genes, encoding the macrolide phosphotransferase C and the ABC transporter MsrA, respectively, were highly prevalent in *S. equorum* and *S. arlettae*, two of the species with the highest estimated prevalence of erythromycin resistance. Prevalence of several resistance determinants that were detected in at least one NAS, is available as a Supplementary File (Table S3).

**Table 2:** Prevalence (and 95% confidence interval) of antimicrobial resistance determinants (ARDs) per isolate associated with various antimicrobial classes in the 3 most frequently isolated bovine non-*aureus* staphylococci (NAS) species.

**Antimicrobial class**	**ARD**	**NAS species^a^**			
		***S. chromogenes***	***S. simulans***	***S. xylosus***	**All NAS**
Aminoglycosides	*aac(6′)*	0 (0–0.06)	0 (0–0.09)	0 (0–0.16)	0 (0–0.01)
	*ant(3″)*	0.03 (0–0.11)	0 (0–0.09)	0 (0–0.16)	0 (0–0.05)
	*ant(4′)*	0.02 (0–0.09)	0 (0–0.09)	0 (0–0.16)	0 (0–0.02)
	*ant(6)*	0.03 (0–0.11)	0 (0–0.09)	0 (0–0.16)	0.01 (0–0.04)
	*aph(3′)*	0 (0–0.06)	0 (0–0.09)	0 (0–0.16)	0.01 (0–0.03)
	*spd*	0 (0–0.06)	0 (0–0.09)	0 (0–0.16)	0 (0–0.02)
Amphenicols	*fexA*	0.02 (0–0.09)	0.05 (0.01–0.18)	0 (0–0.16)	0.01 (0–0.02)
β-Lactams	*blaZ*	0.10 (0.04–0.20)	0 (0–0.09)	0 (0–0.16)	0.06 (0.03–0.11)
	*mecA*	0 (0–0.06)	0 (0–0.09)	0 (0–0.16)	0.01 (0–0.02)^g^
Fluoroquinolones	*parC*^d^	0 (0–0.06)	0 (0–0.09)	0 (0–0.16)	0 (0–0.01)
	*parE*^d^	0 (0–0.06)	0 (0–0.09)	0 (0–0.16)	0 (0–0.01)
Lipopeptides	*Cls*^d^	0 (0–0.06)	0.11 (0.03–0.25)	0 (0–0.16)	0 (0–0.09)
	*mprF*^e^	0 (0–0.06)	1.00 (0.91–1.00)	0 (0–0.16)	0.21 (0.16–0.29)
MDR Efflux Pumps	DHA fam.^f^	0 (0–0.06)	0.05 (0.01–0.18)	1.00 (0.84–1.00)	0.44 (0.36–0.52)
	*mepA*	0 (0–0.06)	0 (0–0.09)	0 (0–0.16)	0.12 (0.08–0.17)
	*norA*	1.00 (0.94–1.00)	1.00 (0.91–1.00)	1.00 (0.84–1.00)	0.96 (0.93–0.98)
	*norB*	0 (0–0.06)	0 (0–0.09)	1.00 (0.84–1.00)	0.29 (0.21–0.37)
	Sav1866	1.00 (0.94–1.00)	1.00 (0.91–1.00)	1.00 (0–1.00)	1.00 (0.91–1.00)
MLS^b^	*ermA*	0 (0–0.06)	0 (0–0.09)	0 (0–0.16)	0 (0–0.01)
	*ermC*	0 (0–0.06)	0 (0–0.09)	0 (0–0.16)	0 (0–0.01)
	*ermT*	0.03 (0–0.11)	0 (0–0.09)	0 (0–0.16)	0.01 (0–0.03)
	*mphC*	0 (0–0.06)	0 (0–0.09)	0.33 (0.17–0.55)	0.10 (0.07–0.15)
	*msrA*	0 (0–0.06)	0 (0–0.09)	0.14 (0.03–0.36)	0.05 (0.03–0.09)
	*vga*	0.03 (0–0.11)	0.03 (0–0.14)	0 (0–0.16)	0.01 (0–0.05)
	*vgbB*	0 (0–0.06)	0 (0–0.09)	0.05 (0–0.24)	0 (0–0.01)
QAC^c^	*qacAB*	0 (0–0.06)	0 (0–0.09)	0 (0–0.16)	0.01 (0–0.02)
Tetracyclines	*tet38*	1.00 (0.94–1.00)	0 (0–0.09)	0 (0–0.16)	0.30 (0.19–0.41)
	*tetK*	0.02 (0–0.09)	0.03 (0–0.14)	0.19 (0.05–0.42)	0.04 (0.02–0.08)
	*tetL*	0.03 (0–0.11)	0.03 (0–0.14)	0 (0–0.16)	0 (0–0.04)
	*tetM*	0 (0–0.06)	0.03 (0–0.14)	0 (0–0.16)	0 (0–0.02)

a *All NAS, all NAS grouped*.

b *Macrolides, lincosamides and streptogramins*.

c *Quaternary ammonium compounds*.

d *Specific substitution in the deduced amino acid sequence (parC = P144S; parE = N404S; cls = T33N)*.

e *Any substitution in the deduced amino acid sequence previously associated with AMR*.

f *DHA family of MFS transporters*.

g *Prevalence estimation ignoring mecA variants*.

### Gene patterns

**Figure 2:**
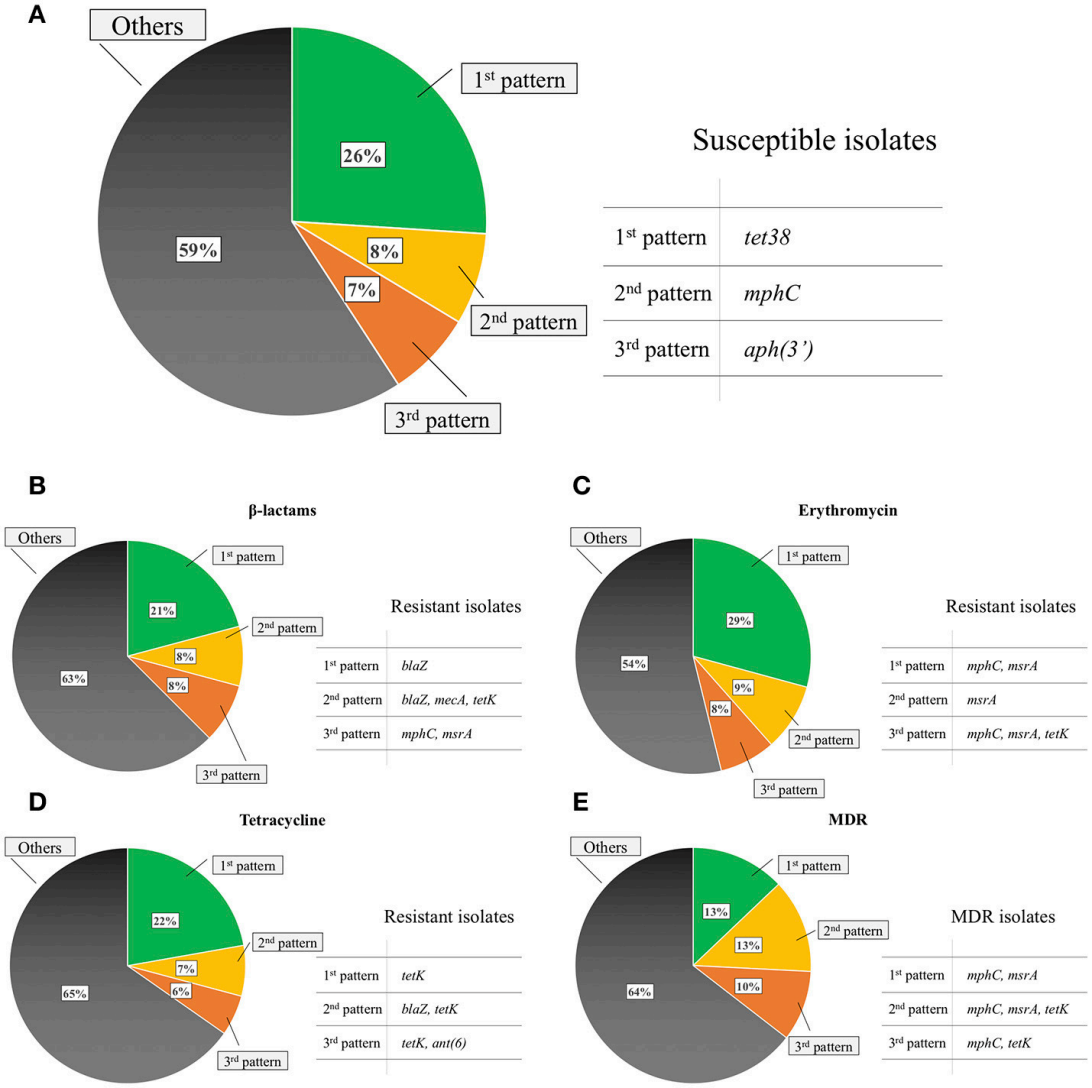
Common gene patterns in susceptible non-*aureus* staphylococci isolates **(A)**, isolates resistant to β-lactams **(B)**, erythromycin **(C)**, tetracycline **(D)**, and in multidrug resistant (MDR) isolates **(E)** when excluding any MDR efflux pumps and antimicrobial resistance-associated residues in the deduced amino acid sequence of any gene evaluated.

The most commonly observed gene patterns were the concomitant presence of *norA, sav1866, tet38* and *folP* AMR-associated residues (51 isolates; 13%), presence of *dha, norA, norB, sav1866*, and *folP* and *gyrB* AMR-associated residues (49 isolates; 12%), and presence of *norA, sav1866*, and *folP* and *mprF* AMR-associated residues (27 isolates; 7%). Gene patterns commonly observed for resistant isolates when excluding MDR efflux pumps and AMR-associated residues included the single presence of the *blaZ* gene (21% of β-lactam-resistant isolates), *tetK* gene (22% of tetracycline-resistant isolates), concomitant presence of *mphC* and *msrA* (29% of erythromycin-resistant isolates) and the same pattern for MDR isolates (13% of MDR isolates; Figure 2).

### Association between resistance determinants and AMR

#### β-lactams

The *blaZ* and *mecA* genes were strongly associated with β-lactam resistance (*P* < 0.001; Table 3). The *blaZ* gene was detected in all *mecA*-positive *S. epidermidis*. The *mecA* variants (*mecA1, mecA sf*) were not associated with β-lactam resistance.

**Table 3:** Relative frequency of drug-specific resistance in bovine non-*aureus* staphylococci isolates conditional on the presence or absence (% Resist | Gene status) of a particular antimicrobial resistance determinant (ARD).

**ARD**	**Resistance^a^**	**Resistance (%)**		***P*-value**
		**Gene absent**	**Gene present**	
*blaZ*	β-Lactams	6.4	65.0	<0.001
*mecA*	β-Lactams	11.6	100	<0.001
*fexA*	Chloramphenicol	6.1	100	<0.001
*cls* T33N	Daptomycin	0.5	0	1
*mprF* G61V	Daptomycin	0	7.7	0.005
*mprF* I420L	Daptomycin	0.7	0	1
*ermA*	Erythromycin	16.5	100	0.005
*ermC*	Erythromycin	16.0	100	0.001
*ermT*	Erythromycin	15.8	100	<0.001
*mphC*	Erythromycin	10.1	49.3	<0.001
*msrA*	Erythromycin	8.6	97.2	<0.001
DHA fam.^b^	MDR^c^	7.0	8.8	0.81
*mepA*	MDR^c^	9.5	1.4	0.16
*norA*	MDR^c^	0	8.9	0.06
*norB*	MDR^c^	9.1	6.0	0.27
SAV1866	MDR^c^	0	8.1	1
*vga*	SYN^d^	6.3	0	1
*tet38*	Tetracycline	19.7	15.1	0.50
*tetK*	Tetracycline	7.7	95.8	<0.001
*tetL*	Tetracycline	16.1	100	<0.001
*tetM*	Tetracycline	18.3	100	0.03

a *Resistance usually associated with the presence of the respective mechanism*.

b *DHA family of MFS transporters*.

c *Multidrug resistant isolates*.

d *Quinupristin/dalfopristin combination*.

#### Amphenicols

Presence of the chloramphenicol/florfenicol MFS efflux transporter FexA always coincided with chloramphenicol resistance. If the *fexA* gene was absent, only 6.1% of isolates were chloramphenicol-resistant (Table 3).

#### Lipopeptides

**Figure 3:**
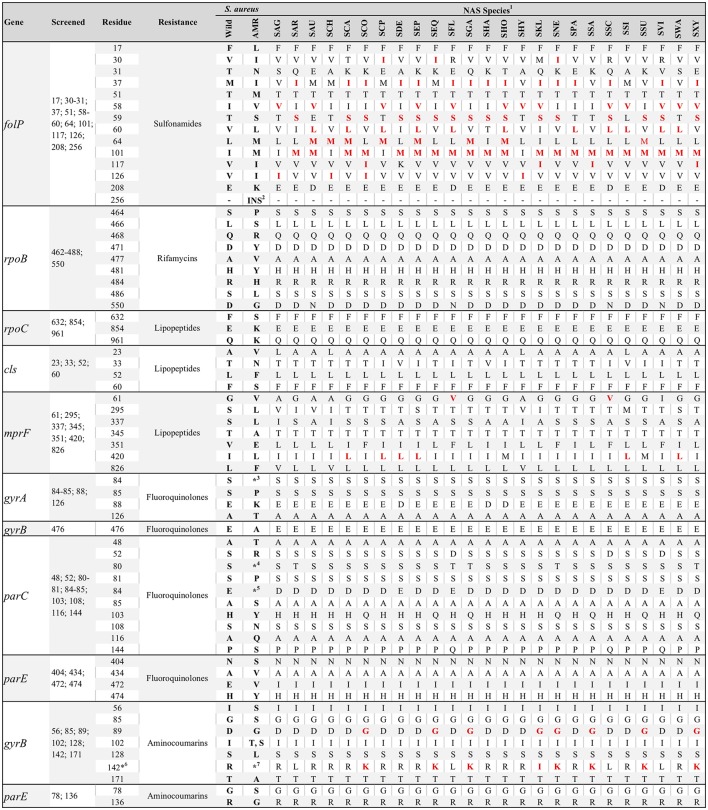
Consensus of residue composition of genes associated with drug-specific antimicrobial resistance for various non-*aureus* staphylococci species (NAS). Positions screened per gene deduced amino acid sequences, in addition to *S. aureus* wild and resistance-associated residues, are also presented. Presence of a red residue for a particular species indicates residues associated with antimicrobial resistance in *Staphylococcus* spp., according to the literature. ^1^SAG, *S. agnetis*; SAR, *S. arlettae*; SAU, *S. auricularis*; SCA, *S. capitis*; SCP, *S. caprae*; SCH, *S. chromogenes*; SCO, *S. cohnii*; SDE, *S. devriesei*; SEP, *S. epidermidis*; SEQ, *S. equorum*; SFL, *S. fleuretti*; SGA, *S. gallinarum*; SHA, *S. haemolyticus*; SHO, *S. hominis*; SHY, *S. hyicus*; SKL, *S. kloosii*; SNE, *S. nepalensis*; SPA, *S. pasteuri*; SSA, *S. saprophyticus*; SSC, *S. sciuri*; SSI, *S. simulans*; SSU, *S. succinus*; SVI, *S. vitulinus*; SWA, *S. warneri*; SXY, *S. xylosus*; ^2^Two-residues insertion at position 256; ^3^A, F, I, L, V, or Y; ^4^F, I, L, V, or Y; ^5^A, G, K, N, V, or Y; ^6^Position 140 for NAS; 142 for *S. aureus*; ^7^K, I or S.

Five *S. sciuri* were resistant to daptomycin, of which two were sequenced. The presence of a valine instead of a glycine at position 61 of the deduced amino acid sequence of the *mprF* gene was present for all *S. sciuri* and *S. fleuretti* (Figure 3). Hence, presence of this residue and daptomycin resistance were associated (*P* = 0.005; Table 3). Leucine instead of an isoleucine at position 420 of the same gene was conserved for some species (Figure 3), and not associated with resistance against daptomycin. A single substitution in the cardiolipin synthase (*cls*) deduced amino acid sequence was observed for five *S. simulans* (T33N) and was not associated with AMR. No residue substitution was detected when contrasting the *cls* deduced amino acid sequence from susceptible versus resistant *S. sciuri*. One daptomycin-resistant *S. sciuri* had a glutamine instead of a lysine at position 711 of the *rpoC* deduced amino acid sequence (K711Q). The same isolate had several substitutions in the deduced amino acid sequence of *mprF* gene, including N31S, I167F, A315V, T331N, N352D, V410I, T460I, E525Q, and D697E (positions based on NCBI's protein accession number ORI05006.1).

#### Macrolides, lincosamides and streptogramins (MLS)

RNA-methyltransferases were associated with erythromycin resistance (*P* < 0.01). Although their presence always coincided with resistance against erythromycin, they were not present in the most common erythromycin-resistant patterns (Figure 2), as their prevalence was relatively low (< 1%; Table 2). In contrast, the *mphC* gene, which encodes the macrolide phosphotransferase C, although associated with erythromycin resistance, was also commonly observed in susceptible isolates, as well as in two of the three most common erythromycin resistance patterns (Figure 2). The efflux pump coding *msrA* gene was strongly associated with AMR (*P* < 0.001). In that regard, 97.2% of the isolates that harbored this determinant were considered erythromycin-resistant, whereas only 8.6% of isolates that did not have the same element were resistant. The concomitant presence of *emr, msrA*, and *mphC* was not detected in the present study. No interaction was observed between *msrA* and *mphC*. The final model contained estimates for both *msrA* ($\hat{\beta }$ = 4.34, SE = 0.93, *P* < 0.001) and *mphC* ($\hat{\beta }$ = 1.46, SE = 0.30, *P* < 0.001). The estimated odds ratio of erythromycin resistance associated with the *msrA* gene was 76.7 (95% CI 12.4–475) and 4.3 for *mphC* (95% CI 2.4–7.8), irrespective of the presence of the other. There was no association between the *vga* gene, which encodes a streptogramin A ABC transporter, and quinupristin/dalfopristin resistance (*P* = 1.00; Table 3).

#### Tetracyclines

*tet38* was commonly detected in susceptible isolates (Figure 2). Other tetracycline efflux protein genes detected included *tetK* and *tetL*, both associated with tetracycline resistance (*P* < 0.001; Table 3), as well as *tetM*, which encodes for a tetracycline ribosomal protection protein. *tetM* was only present in two tetracycline-resistant *S. simulans* isolates. *tetK* and *tetL* were not together in the same isolate.

#### Other antimicrobials

A single *S. haemolyticus* isolate was resistant to rifamycins but its sequence was not available. Intrinsically novobiocin-resistant NAS species such as *S. sciuri, S. vitulinus, S. fleuretti, S. saprophyticus, S. cohnii, S. equorum, S. kloosii, S. arlettae, S. gallinarum, S. nepalensis, S. succinus*, and *S. xylosus*, harbored residues other than arginine at the *gyrB* deduced amino acid sequence position 142 (140 for NAS); either a lysine (*S. saprophyticus, S. cohnii, S. equorum, S. gallinarum, S. nepalensis, S. succinus* and *S. xylosus*) or a leucine (*S. sciuri, S. vitulinus, S. arlettae*, and *S. fleuretti*). The single *S. kloosii* had an isoleucine at position 140 (Figure 3). MDR efflux pump genes were not associated with the MDR phenotype (Table 3).

## Discussion

In this study, the prevalence of AMR and corresponding resistance determinants in NAS isolated from dairy herds were estimated. Moreover, we determined whether resistance determinants were associated with drug-specific AMR. The relatively large sample size from 89 herds allowed estimation of the prevalence of genetic elements not limited to resistant isolates, and the diversity of species allowed searching for species-specific resistance mechanisms. Resistance to vancomycin, fluoroquinolones, linezolid and daptomycin was absent or uncommon. These drugs are considered critically important in human medicine and their use is restricted in food-producing animals in several countries. Resistance against highly important antimicrobials, frequently used in dairy herds (e.g., penicillins and tetracyclines) was relatively common. Some NAS species had species-specific patterns of resistance against specific antimicrobials. Moreover, prevalence of AMR genetic determinants was also species-specific; for example, the prevalence of the *mecA* elements was estimated to be 17% in *S. epidermidis*, but close to zero for other species isolated from bovine milk. It was also clear that some genetic determinants were involved in drug-specific AMR, whereas others were not. Finally, this study used a novel approach that combined bioinformatics tools with advanced statistical methods to estimate the true prevalence of resistance determinants, highlighting the importance of tools such as WGS for AMR studies.

It is estimated that, without urgent action, we are heading toward a post-antimicrobial era where 700,000 human deaths per year will be attributable to AMR (O'Neill, 2016). NAS account for up to 30% of all clinically relevant blood-stream infections in humans (Piette and Verschraegen, 2009). In comparison to nosocomial NAS isolates, where resistance to glycopeptides, fluoroquinolones, lincosamides, etc., is common, drastically limiting therapeutic options (May et al., 2014), presence of AMR was relatively uncommon for bovine isolates. Nevertheless, based on experience in human medicine, NAS are pathogens that potentially acquire resistance mechanisms and virulence genes, and are able to persist in presence of intense antimicrobial use. Irrespective of the apparent zoonotic potential that NAS isolates obtained from mastitic milk may have (Thorberg et al., 2006), NAS of animal origin are believed to be important reservoirs of ARGs, which is of utmost importance for human and veterinary medicine. Indeed, the *mecA* gene was frequently detected in *S. epidermidis*, the most common staphylococcal species recovered from humans (Becker et al., 2014).

Results for NAS (or CNS) were historically reported as a group. However, it recently became clear that these species should be treated individually, as risk factors can be species-specific (De Visscher et al., 2017). In our study, prevalence of AMR was clearly species-dependent. Some extreme cases were observed, such as streptogramins resistance for *S. gallinarum*, erythromycin resistance in *S. cohnii*, and erythromycin and penicillin resistance for *S. arlettae*. It is noteworthy that *S. gallinarum* is frequently isolated from poultry (Shi et al., 2015) but only occasionally infects dairy cows. In Canada, virginiamycin is used in poultry for growth promotion and prevention of infectious diseases. Its use has been associated with AMR (Aarestrup et al., 2001), and a high prevalence of streptogramin resistance in *Enterococcus faecium* was reported from Canadian poultry (Diarra et al., 2010). Based on our results, we hypothesized that *S. gallinarum* isolated from Canadian dairy cows carried resistance mechanisms that were developed/selected in response to the use of virginiamycin in poultry. Since no streptogramin-related resistance determinant was observed, including *erm, vgb, msr, vga, vat, lsa* genes among others, we were not able to make any inferences regarding the genetic basis of streptogramin resistance in NAS that could explain our findings. For *S. arlettae*, there was a high prevalence of erythromycin and penicillin resistance. Since the estimated prevalence of the *mphC* and *msrA* genes for this species was high, macrolide resistance was expected. However, we were not able to determine genetic mechanisms involved in penicillin resistance for *S. arlettae*. Perhaps recently discovered β-lactam resistance mechanisms (Andreis et al., 2017) other than those screened, were involved in penicillin resistance for *S. arlettae*. Preliminary screening for the recently described *bla*_ARL_ gene (GenBank APY23733.1) returned perfect hits for all *S. arlettae* sequenced in the present study, indicating that besides being highly prevalent in this species, this gene might confer β-lactam resistance in *S. arlettae* isolated from Canadian dairy herds (results not shown).

NAS and other Gram-positive bacteria are intrinsically resistant to aztreonam, temocillin, polymyxin B, colistin, and nalidixic acid (European Committee on Antimicrobial Susceptibility Testing, 2016). Noteworthy, despite third-generation cephalosporins routinely being employed as treatment of staphylococcal infections in animals including mastitis, NAS are intrinsically resistant to ceftazidime (Wiktorowicz-Belzyt et al., 1997; European Committee on Antimicrobial Susceptibility Testing, 2016). In addition, species such as *S. saprophyticus, S. cohnii*, and *S. xylosus* share an intrinsic resistance to novobiocin, whereas specific resistance patterns are also known to exist (e.g., *S. saprophyticus* resistance to fusidic acid and fosfomycin, and *S. capitis* resistance to fosfomycin) (Clinical Laboratory Standards Institute, 2016). Although it is well-established that acquired resistance to β-lactams, tetracyclines, aminoglycosides, macrolides, lincosamides and chloramphenicol may occur in bovine NAS isolates (Oliver and Murinda, 2012; Klimiene et al., 2016), phenotypic AMR patterns observed suggest that intrinsic mechanisms of AMR might be present for a subset of NAS species. The most conceivable hypothesis is the presence of an intrinsic erythromycin resistance in *S. arlettae*. High-level erythromycin resistance in *S. arlettae* was reported (Lüthje and Schwarz, 2006), although the relatively low prevalence of this species limited further assessments. Here, we first observed this behavior in a relatively large number of isolates obtained from several geographical locations, and also elucidated genetic mechanisms that are likely to explain these findings.

Acquired ARGs are usually present within mobile DNA that is capable to move from one genome to another and also within a genome. The most prominent example are plasmid-encoded ARGs, which encompass resistance mechanisms to almost all classes of antimicrobials available (Bennett, 2008). Genes encoding aminoglycosides modifying enzymes detected in the present study are mostly often plasmid-encoded (Mingeot-Leclercq et al., 1999), which supports its role in drug-specific acquired resistance. *blaZ*-mediated penicillinase, the most prevalent mechanism of penicillin resistance in NAS, is on mobile elements of which the majority are chromosomally located for bovine *Staphylococcus* spp. isolates (Olsen et al., 2006). In terms of tetracycline resistance, *tetK* and *tetL* are generally present on small plasmids that may integrate into chromosomal DNA in *S. aureus* (Chopra and Roberts, 2001). Genes encoding efflux pumps can be present on plasmids (e.g., *qacAB*). However, their location on the chromosome followed by over-expression provides the bacterium an intrinsic resistance mechanism, without the need of new genetic material (Webber and Piddock, 2003). In our study, *norA, norB, sav1866, mepA* and *msrA* are known to be chromosomally located (Costa et al., 2013).

Presence of resistance determinants is frequently associated with AMR (Hu et al., 2017). In NAS, *bla*Z, *mecA, fexA, erm, mphC, msrA*, and *tet* genes were involved in drug-specific AMR. The most commonly observed patterns of acquired resistance mechanisms were that a subset of these elements was highly prevalent in resistant isolates, but not common in susceptible ones. Taken together, we inferred that when excluding MDR efflux pumps and any mutations in the deduced amino acid sequence of the *folP, gyrB*, and *mprF* genes, three elements (*blaZ, msrA*, and *tetK*) were crucial for the AMR-phenotype in NAS isolated from Canadian dairy herds. Their presence was followed by drug-specific AMR; however, in their absence, AMR was uncommon. Both *blaZ* and *tetK* were highly prevalent in *S. epidermidis*, whereas *msrA* was common in *S. arlettae, S. cohnii*, and *S. equorum*. These genes have been detected in NAS isolated from dairy cows (Bagcigil et al., 2012; Li et al., 2015; Bochniarz et al., 2016). Their dissemination undermines therapeutic options, since β-lactams, macrolides and tetracyclines are among the most commonly used antimicrobials in Canadian dairy herds (Saini et al., 2012a). Whether the prevalence of these elements is related to the use of antimicrobials in dairy cattle is yet to be determined. Of note, the majority of Gram-negative isolates usually carries a single type of *tet* gene, which was also observed for our Gram-positive NAS isolates (Chopra and Roberts, 2001).

MDR efflux pumps were observed for almost all isolates, corroborating previous findings (Antiabong et al., 2017). In general, efflux pumps need to be up-regulated to be associated with AMR; furthermore, several factors can have an impact on this regulation. Hence, their presence does not necessarily translate into the presence of AMR. Regulation of MDR efflux pumps is complex, with several regulators involved in the expression of these elements (Costa et al., 2013). Gene expression assays are required to determine the true impact of these elements in AMR. Still, some elements, e.g., the *norA* gene, are known to be associated with a low level of reduced susceptibility against fluoroquinolones (Kaatz and Seo, 1995). Therefore, use of a clinical breakpoint to define a resistant profile is likely to miss the true association between presence of these elements and AMR, especially if the increase in MIC resulting from their presence is relatively small. In the present study, no MDR efflux pump was associated with presence of the MDR phenotype, although the high prevalence of these elements in NAS was cause for concern.

Residues in the deduced amino acid sequence of the *folP* and *mprF* genes previously associated with AMR were common in NAS isolates. To our knowledge, this was the first study to screen for *folP* and *mprF* AMR-associated residues in NAS. Previous mutations resulting in the presence of these residues were described for *S. aureus* exclusively (Hampele et al., 1997; Friedman et al., 2006; Bayer et al., 2013), and there is the inherent risk of concluding that these mechanisms are associated with AMR in NAS because they are associated with AMR in *S. aureus*. Because no sulfonamide was included in the MIC test (only the combination of trimethoprim and sulfamethoxazole was tested), the role of *folP* mutations in sulfonamide resistance for NAS could not be determined. However, the estimated mean number of *folP* AMR-associated residues was higher in isolates resistant to the combination of trimethoprim and sulfamethoxazole than in susceptible isolates (5 vs. 3.75 residues respectively, results not shown). The *mprF* gene product, multipeptide resistance factor (MprF), is responsible for lysinylation of the phosphotidylglycerol and translocation of the same to the cell membrane, which results in a positively charged cell membrane. This membrane repels the active form of daptomycin (calcium-daptomycin), a cationic peptide (Bayer et al., 2013) reducing its efficacy. It is hypothesized that non-synonymous mutations in the *mprF* gene are responsible for a gain-of-function of the same. One specific substitution (G61V) was present on its deduced amino acid sequence for all *S. sciuri*, the only species resistant to daptomycin. If *S. sciuri* has a more positively charged cell membrane than other species, as a consequence of this or any other *mprF* mutation observed, this species could be less sensitive to daptomycin. In this study, we provided a list of candidate substitutions in the deduced amino acid sequences of the *rpoC, mprF* and *cls* genes that could be associated with daptomycin resistance in NAS. The availability of species-specific consensus sequences for key residues should aid in elucidating mechanisms of sulfonamide and lipopeptide resistance.

This study was apparently the first to report consensus protein sequences for the QRDR of 25 NAS species. *gyrA* residue 88 and *parC* residues 80 and 84 had species-specific compositions, as previously reported for a subset of NAS species (Linde et al., 2001). *parC* and *parE* mutations previously associated with increased MIC were rarely detected, except for *S. devriesei*, where the P144S substitution in the *parC* gene deduced amino acid sequence was relatively common. The role of each specific substitution observed toward fluoroquinolones resistance was not evaluated, as no NAS was resistant to fluoroquinolones (based on clinical breakpoints). Studies using a wider range of fluoroquinolones concentrations where the role of each substitution could be carefully evaluated would be useful to establish a causal association between specific substitution and AMR. Following the same logic, NAS isolated from species where the use of fluoroquinolones is higher would enhance representativeness of resistant isolates, since the estimated prevalence of fluoroquinolones resistance and associated mechanisms in NAS isolated from dairy cows was low. This result was somewhat expected, as QRDR mutations are a consequence of prolonged exposure to fluoroquinolones (Roychoudhury et al., 2001) and relative use of these antimicrobials in the Canadian dairy industry is low (Saini et al., 2012a).

Aminocoumarins are infrequently used in the dairy industry, except when combined with a β-lactam such as penicillin (Saini et al., 2012a). Novobiocin is commonly used for identification of NAS, as some species are intrinsically novobiocin-resistant (Schleifer et al., 1984; Cunha et al., 2004). Non-synonymous mutations in the *gyrB* and *parE* genes are associated with novobiocin-resistance in *S. aureus* (Fujimoto-Nakamura et al., 2005), whereas the presence of specific residues in the *gyrB* deduced amino acid sequence explains the intrinsic resistance of some NAS (Vickers et al., 2007). In the present study, novobiocin was tested only in combination with penicillin, because these two compounds are used together in all formulations containing novobiocin that are available for the Canadian dairy industry. This limited our assessment of species-specific mechanisms of novobiocin resistance that could have been evaluated if two populations (resistant and susceptible) were observed, conditional on the species. Nevertheless, all NAS intrinsically resistant to novobiocin harbored either a lysine, leucine or isoleucine at *gyrB* deduced amino acid sequence position 140. Since it is well-established that a lysine at position 140 is responsible for intrinsic resistance in *S. saprophyticus* (Vickers et al., 2007), we hypothesize that the presence of a lysine, leucine or isoleucine at position 140 is responsible for intrinsic novobiocin resistance in *S. sciuri, S. vitulinus, S. fleuretti, S. cohnii, S. equorum, S. kloosii, S. arlettae, S. gallinarum, S. nepalensis, S. succinus*, and *S. xylosus*.

This study demonstrated the importance of WGS in AMR studies. The presence of some resistance determinants was strongly associated with drug-specific AMR; furthermore, some specific mutations were probably the basis of intrinsic resistance. Hence, as sequencing technologies become more affordable and available resulting in studies similar to this one, prediction of resistance patterns based on the presence of key elements is achievable. For example, an infection caused by a NAS that harbors the *msrA* gene is likely to be unresponsive to erythromycin, assuming that *in vivo* results mimic those *in vitro*. Furthermore, WGS has the added benefit of species identification and classification (Mellmann et al., 2006; Naushad and Gupta, 2013). In addition, WGS can be done substantially faster than bacteriological culture followed by additional biochemical tests, and the availability of species identification provides an immeasurable advantage to WGS when contrasting it to standard laboratory techniques used in AMR studies. Species identification can provide insights regarding the likely outcome of an antimicrobial therapy. For example, the outcome of an antimicrobial therapy is likely to be different for an IMI caused by *S. arlettae* and the same caused by *S. chromogenes* (again assuming that *in vivo* results mimic the *in vitro* ones).

The dairy industry has undergone considerable changes over the last decade (Barkema et al., 2015). Antimicrobial use will be impacted by two relatively recent trends: (1) adoption of selective instead of blanket dry cow therapy, and (2) transition to organic farming practices. Because there is a well-established association between antimicrobial use and resistance, NAS isolated from herds that transitioned to organic or adopted the use of selective dry-cow therapy can be less resistant to antimicrobials following reduced antimicrobial use (Park et al., 2012). Yet, we believe that our results are still valid for the contemporary dairy industry, irrespective of both trends, for at least three reasons: First, the proportion of organic farms in Canada increased only slightly, from 1% in 2007 to 2% in 2016 (Canadian Dairy Information Centre, 2017), especially compared to European countries (Barkema et al., 2015). Secondly, the Canadian dairy industry has not yet adopted the use of selective dry cow therapy. Moreover, decreased antimicrobial use due to introduction of selective dry cow therapy might not affect the prevalence of AMR in NAS at all, based on preliminary results (Nobrega et al., submitted). Finally, the antimicrobial formulations available for the dairy industry did not change substantially over the last decade; essentially, the same classes of antimicrobials are still in use. Thus, drastic changes in prevalence of AMR in mastitis pathogens are not expected. In conclusion, under the assumption that other factors related to total antimicrobial use remained relatively constant for Canadian dairy industry over the last decade, our prevalence estimates should still be applicable to the modern dairy industry.

Our study had some limitations. First, we based all our prevalence estimation on samples from 89 dairy herds enrolled in a milk quality program (DHI). As herds not enrolled were not sampled, our estimates could be biased if the prevalence of AMR and resistance determinants in NAS depended on extraneous factors associated with enrolment (i.e., enrolled herds used more antimicrobials than non-enrolled herds, or vice-versa). Secondly, the absence of specific drugs (novobiocin, sulfonamides), outcomes (fluoroquinolones, vancomycin, linezolid resistance), broader range of MIC values (fluoroquinolones), isolates per species (*S. caprae, S. auricularis, S. kloosii, S. fleuretti, S. nepalensis, S. hyicus*), and sufficient number of resistant isolates (daptomycin and rifamycin), prevented us from further assessments of resistance mechanisms, including species-specific ones. Another limitation was that our genetic results depended on the queries used. As the number of species-specific queries increases in databases (i.e., *bla*_ALR_ for *S*. *arlettae*), the sensitivity of our methodology also increases, as well as the likelihood of finding resistance mechanisms. Moreover, the estimated prevalence of resistance determinants could also decrease significantly. For example, a query for the *norA* gene, a MFS efflux pump, could return high quality hits for a second MFS efflux pumps other than NorA. If at a later time, queries for such hypothetical second efflux pump become available, the estimated prevalence of the *norA* gene will decrease, because a region in the genome could only map to a single query in our methodology. Nevertheless, all returned hits were confirmed by BLASTp searches against NCBI's *nr* database. Although this did not eliminate the risk of false-positives, it provided an opportunity to confirm the original hits and exclude hits closely related to intrinsic components of the bacteria not associated with AMR. Similarly, more queries representing more resistance mechanisms could identify resistance determinants that we likely missed, such as those associated with streptogramins resistance in *S. gallinarum*. A limitation of our analyses regarding the association between the presence of ARGs and AMR was the loss of substantial information when dichotomizing the MIC results into resistant and susceptible isolates. The presence of a gene or residue might not be, by itself, enough to increase the MIC above the CLSI clinical threshold for defining resistance. Instead, the same element might be associated with a small increase in the MIC, enhancing the ability of the bacteria to survive in the presence of the antimicrobial and facilitating acquisition of other genetic elements. Similarly, MIC measurements are known to suffer from variability, characterized by a variation of one 2-fold dilution from its true value (Brown and Traczewski, 2009). In that regard, isolates with true MIC value close to or at the breakpoints (e.g., isolates with intermediate resistance harboring a resistance mechanism) can suffer from misclassification bias, where associations between presence of gene and resistance would be underestimated. Hence, analyses based on the actual MICs that were obtained from a broad range of dilutions should provide better estimates of true associations between ARGs and AMR in NAS. Finally, residue substitutions in *S. aureus* that are associated with AMR might not have the same effect in NAS.

In summary, prevalence of AMR in NAS isolated from Canadian dairy herds was higher for tetracycline, penicillin and erythromycin compared to other antimicrobials, and was NAS species-dependent. *S. arlettae* had the highest prevalence of MDR. No isolate was resistant to vancomycin, linezolid or fluoroquinolones. The most frequently identified resistance determinants were mutations in the *folP* gene and MDR efflux pumps, which were present in all isolates and not associated with the MDR phenotype. The *blaZ, mecA, fexA, erm, mphC, msrA*, and *tet* genes were associated with drug-specific AMR. There were specific residues in *gyrB* for NAS species intrinsically resistant to novobiocin. Finally, this study illustrated the importance of WGS in AMR studies.

## Author contributions

DN, VS, CL, JK, JD, and HB: conceived and designed the study; DN, LC, and SAN: performed laboratory activities; DN and SN: worked on the sequencing data; DN and SAN: performed statistical analyses; DN: wrote the first draft of the manuscript; JK, CL, VS, JD, and HB: revised the document for important intellectual content. All authors gave approval of the final version to be published and agreed to be accountable for all aspects of the work.

### Conflict of interest statement

The authors declare that the research was conducted in the absence of any commercial or financial relationships that could be construed as a potential conflict of interest.
