# Supplementary material for: Prevalence and Genetic Basis of Antimicrobial Resistance in Non-aureus Staphylococci Isolated from Canadian Dairy Herds
Source: Front Microbiol. 2018 Feb 16;9:256. doi: 10.3389/fmicb.2018.00256 (PMC5820348; doi:10.3389/fmicb.2018.00256)
Supplement: Supplementary file 1 [file Table1.DOCX]

Supplementary Material

**Prevalence and Genetic Basis of Antimicrobial Resistance in Non-*aureus* Staphylococci Isolated from Canadian Dairy Herds**

**Diego B. Nobrega*^1,2^, Sohail Naushad^1,2^, S. Ali Naqvi^1,2^, Larissa A. Z. Condas^1,2^, Vineet Saini^1,2,3^, John P. Kastelic^1^, Christopher Luby^2,4^, Jeroen De Buck^1,2^, and Herman W. Barkema^1,2,5^**

**^*^Correspondence:**

Dr. Diego B Nobrega

[diego.nobrega@ucalgary.ca](mailto:diego.nobrega@ucalgary.ca)

| **Table S1.** Clinical breakpoints used for categorization of non-*aureus* staphylococci isolates into susceptible or resistant for the antimicrobials evaluated. | | |
| --- | --- | --- |
| Antimicrobial | Breakpoint^1^ | Source^2^ |
| Ampicillin | 0.5 μg/mL | CLSI, 2008 |
| Chloramphenicol | 16 μg/mL | CLSI, 2016 |
| Ceftiofur | 4 μg/mL | CLSI, 2008^3^ |
| Cephalotin | 16 μg/mL | CLSI, 2008 |
| Ciprofloxacin | 2 μg/mL | CLSI, 2016 |
| Clindamycin | 1 μg/mL | CLSI, 2016 |
| Daptomycin | 2 μg/mL | CLSI, 2016 |
| Erythromycin | 1 μg/mL | CLSI, 2016 |
| Gentamicin | 8 μg/mL | CLSI, 2016 |
| Levofloxacin | 2 μg/mL | CLSI, 2016 |
| Linezolid | 8 μg/mL | CLSI, 2016 |
| Moxifloxacin | 1 μg/mL | CLSI, 2016 |
| Nitrofurantoin | 64 μg/mL | CLSI, 2016 |
| Oxacillin | 4 μg/mL | CLSI, 2016^3^ |
| Penicillin | 0.25 μg/mL | CLSI, 2016 |
| Penicillin/novobiocin | 2/4 μg/mL | CLSI, 2008^3^ |
| Pirlimycin | 4 μg/mL | CLSI, 2008^3^ |
| Quinupristin/dalfopristin | 2 μg/mL | CLSI, 2016 |
| Rifampin | 2 μg/mL | CLSI, 2016 |
| Tetracycline | 8 μg/mL | CLSI, 2016 |
| Tigecycline | 1 mg/L | EUCAST, 2017 |
| Trimethoprim/sulfamethoxazole | 4/76 μg/mL | CLSI, 2016 |
| Vancomycin | 8 μg/mL | CLSI, 2016 |

^1^MIC necessary to classify the isolate as, at least, of intermediate resistance according to literature source.

^2^Clinical and Laboratory Standards Institute (2008). Performance standards for antimicrobial disk and dilution susceptibility tests for bacteria isolated from animals; approved standard - 3^rd^ ed. CLSI document VET01S;

Clinical and Laboratory Standards Institute (2016). Performance standards for antimicrobial susceptibility testing. 26^th^ ed. CLSI document M100-S26.

European Committee on Antimicrobial Susceptibility Testing (2017). Available: *Breakpoint tables for interpretation of MICs and zone diameters, version 7.1*. http://www.eucast.org/fileadmin/src/media/PDFs/EUCAST_files/Breakpoint_tables/v_7.1_Breakpoint_Tables.pdf

^3^*Staphylococcus aureus* breakpoint
