# Supplementary material for: Prevalence and Genetic Basis of Antimicrobial Resistance in Non-aureus Staphylococci Isolated from Canadian Dairy Herds
Source: Front Microbiol. 2018 Feb 16;9:256. doi: 10.3389/fmicb.2018.00256 (PMC5820348; doi:10.3389/fmicb.2018.00256)
Supplement: Supplementary file 2 [file Table2.DOCX]

Supplementary Material

**Prevalence and Genetic Basis of Antimicrobial Resistance in Non-*aureus* Staphylococci Isolated from Canadian Dairy Herds**

**Diego B. Nobrega*^1,2^, Sohail Naushad^1,2^, S. Ali Naqvi^1,2^, Larissa A. Z. Condas^1,2^, Vineet Saini^1,2,3^, John P. Kastelic^1^, Christopher Luby^2,4^, Jeroen De Buck^1,2^, and Herman W. Barkema^1,2,5^**

**^*^Correspondence:**

Dr. Diego B Nobrega

[diego.nobrega@ucalgary.ca](mailto:diego.nobrega@ucalgary.ca)

**Table S2.** MIC_50_, MIC_90_ and range of MIC values (μg/mL) observed according to antimicrobial and non-*aureus* staphylococci (NAS) species.

| Species | AMP^1^ | | |  | CEF | | |  | CEP | | |
| --- | --- | --- | --- | --- | --- | --- | --- | --- | --- | --- | --- |
|  | MIC_50_ | MIC_90_ | Range |  | MIC_50_ | MIC_90_ | Range |  | MIC_50_ | MIC_90_ | Range |
| *S. chromogenes* | ≤0.12 | 0.5 | ≤0.12 - >8 |  | 1 | 1 | ≤0.5 - 4 |  | ≤2 | ≤2 | ≤2 - ≤2 |
| *S. simulans* | ≤0.12 | ≤0.12 | ≤0.12 - 2 |  | ≤0.5 | 1 | ≤0.5 - 2 |  | ≤2 | ≤2 | ≤2 - ≤2 |
| *S. xylosus* | ≤0.12 | ≤0.12 | ≤0.12 - 8 |  | ≤0.5 | 1 | ≤0.5 - 2 |  | ≤2 | ≤2 | ≤2 - ≤2 |
| *S. haemolyticus* | ≤0.12 | 0.25 | ≤0.12 - 1 |  | 1 | 2 | ≤0.5 - 2 |  | ≤2 | ≤2 | ≤2 - ≤2 |
| *S. epidermidis* | ≤0.12 | 0.25 | ≤0.12 - 8 |  | ≤0.5 | 2 | ≤0.5 - 4 |  | ≤2 | ≤2 | ≤2 - ≤2 |
| *S. cohnii* | ≤0.12 | 0.25 | ≤0.12 - 0.25 |  | 1 | 1 | ≤0.5 - 2 |  | ≤2 | ≤2 | ≤2 - ≤2 |
| *S. sciuri* | ≤0.12 | ≤0.12 | ≤0.12 - 0.25 |  | 1 | 2 | ≤0.5 - 2 |  | ≤2 | ≤2 | ≤2 - ≤2 |
| *S. capitis* | ≤0.12 | ≤0.12 | ≤0.12 - 0.25 |  | ≤0.5 | ≤0.5 | ≤0.5 - ≤0.5 |  | ≤2 | ≤2 | ≤2 - ≤2 |
| *S. gallinarum* | ≤0.12 | 0.25 | ≤0.12 - 0.25 |  | 1 | 1 | ≤0.5 - 2 |  | ≤2 | ≤2 | ≤2 - ≤2 |
| *S. warneri* | ≤0.12 | 0.5 | ≤0.12 - 0.5 |  | ≤0.5 | ≤0.5 | ≤0.5 - ≤0.5 |  | ≤2 | ≤2 | ≤2 - ≤2 |
| *S. saprophyticus* | 0.25 | 0.25 | ≤0.12 - 0.25 |  | 1 | 2 | ≤0.5 - 4 |  | ≤2 | ≤2 | ≤2 - ≤2 |
| *S. arlettae* | 0.25 | 0.5 | ≤0.12 - 1 |  | 1 | 2 | ≤0.5 - 2 |  | ≤2 | ≤2 | ≤2 - ≤2 |
| *S. succinus* | ≤0.12 | ≤0.12 | ≤0.12 - 0.25 |  | 1 | 2 | ≤0.5 - 2 |  | ≤2 | ≤2 | ≤2 - ≤2 |
| *S. agnetis* | ≤0.12 | 0.25 | ≤0.12 - 0.25 |  | 1 | 1 | ≤0.5 - 1 |  | ≤2 | ≤2 | ≤2 - ≤2 |
| *S. hominis* | ≤0.12 | ≤0.12 | ≤0.12 - ≤0.12 |  | ≤0.5 | ≤0.5 | ≤0.5 - ≤0.5 |  | ≤2 | ≤2 | ≤2 - ≤2 |
| NAS | ≤0.12 | 0.25 | ≤0.12 - >8 |  | ≤0.5 | 1 | ≤0.5 - 4 |  | ≤2 | ≤2 | ≤2 - ≤2 |
|  |  |  |  |  |  |  |  |  |  |  |  |
| Species | CHL | | |  | CIP | | |  | CLI | | |
|  | MIC_50_ | MIC_90_ | Range |  | MIC_50_ | MIC_90_ | Range |  | MIC_50_ | MIC_90_ | Range |
| *S. chromogenes* | 8 | 8 | ≤2 - >16 |  | ≤1 | ≤1 | ≤1 - ≤1 |  | ≤0.5 | ≤0.5 | ≤0.5 - >2 |
| *S. simulans* | 8 | 8 | ≤2 - >16 |  | ≤1 | ≤1 | ≤1 - ≤1 |  | ≤0.5 | ≤0.5 | ≤0.5 - >2 |
| *S. xylosus* | 8 | 8 | ≤2 - >16 |  | ≤1 | ≤1 | ≤1 - ≤1 |  | ≤0.5 | ≤0.5 | ≤0.5 - >2 |
| *S. haemolyticus* | 4 | 8 | ≤2 - >16 |  | ≤1 | ≤1 | ≤1 - ≤1 |  | ≤0.5 | ≤0.5 | ≤0.5 - >2 |
| *S. epidermidis* | 8 | 8 | ≤2 - 16 |  | ≤1 | ≤1 | ≤1 - ≤1 |  | ≤0.5 | ≤0.5 | ≤0.5 - >2 |
| *S. cohnii* | 8 | 8 | 4 - >16 |  | ≤1 | ≤1 | ≤1 - ≤1 |  | ≤0.5 | ≤0.5 | ≤0.5 - >2 |
| *S. sciuri* | 4 | 8 | ≤2 - >16 |  | ≤1 | ≤1 | ≤1 - ≤1 |  | ≤0.5 | 1 | ≤0.5 - 1 |
| *S. capitis* | 4 | 8 | ≤2 - 8 |  | ≤1 | ≤1 | ≤1 - ≤1 |  | ≤0.5 | ≤0.5 | ≤0.5 - ≤0.5 |
| *S. gallinarum* | 8 | 8 | 4 - 16 |  | ≤1 | ≤1 | ≤1 - ≤1 |  | ≤0.5 | ≤0.5 | ≤0.5 - ≤0.5 |
| *S. warneri* | 8 | 8 | 4 - 8 |  | ≤1 | ≤1 | ≤1 - ≤1 |  | ≤0.5 | 1 | ≤0.5 - >2 |
| *S. saprophyticus* | 8 | 8 | 4 - 8 |  | ≤1 | ≤1 | ≤1 - ≤1 |  | ≤0.5 | ≤0.5 | ≤0.5 - ≤0.5 |
| *S. arlettae* | 8 | 8 | 4 - >16 |  | ≤1 | ≤1 | ≤1 - ≤1 |  | 1 | 2 | ≤0.5 - 2 |
| *S. succinus* | 8 | 8 | 8 - 8 |  | ≤1 | ≤1 | ≤1 - ≤1 |  | ≤0.5 | ≤0.5 | ≤0.5 - ≤0.5 |
| *S. agnetis* | 8 | 8 | 4 - 16 |  | ≤1 | ≤1 | ≤1 - ≤1 |  | ≤0.5 | ≤0.5 | ≤0.5 - ≤0.5 |
| *S. hominis* | 4 | 8 | ≤2 - 8 |  | ≤1 | ≤1 | ≤1 - ≤1 |  | ≤0.5 | ≤0.5 | ≤0.5 - ≤0.5 |
| NAS | 8 | 8 | ≤2 - >16 |  | ≤1 | ≤1 | ≤1 - ≤1 |  | ≤0.5 | ≤0.5 | ≤0.5 - >2 |

**Table S2.** **(cont.)**

| Species | DAP^1^ | | |  | ERY | | |  | GEN | | |
| --- | --- | --- | --- | --- | --- | --- | --- | --- | --- | --- | --- |
|  | MIC_50_ | MIC_90_ | Range |  | MIC_50_ | MIC_90_ | Range |  | MIC_50_ | MIC_90_ | Range |
| *S. chromogenes* | ≤0.5 | ≤0.5 | ≤0.5 - 1 |  | ≤0.25 | 0.5 | ≤0.25 - >4 |  | ≤2 | ≤2 | ≤2 - ≤2 |
| *S. simulans* | ≤0.5 | ≤0.5 | ≤0.5 - ≤0.5 |  | ≤0.25 | 0.5 | ≤0.25 - >4 |  | ≤2 | ≤2 | ≤2 - ≤2 |
| *S. xylosus* | ≤0.5 | ≤0.5 | ≤0.5 - 1 |  | ≤0.25 | >4 | ≤0.25 - >4 |  | ≤2 | ≤2 | ≤2 - ≤2 |
| *S. haemolyticus* | ≤0.5 | ≤0.5 | ≤0.5 - 1 |  | ≤0.25 | ≤0.25 | ≤0.25 - >4 |  | ≤2 | ≤2 | ≤2 - ≤2 |
| *S. epidermidis* | ≤0.5 | ≤0.5 | ≤0.5 - 1 |  | ≤0.25 | 0.5 | ≤0.25 - >4 |  | ≤2 | ≤2 | ≤2 - 4 |
| *S. cohnii* | ≤0.5 | ≤0.5 | ≤0.5 - 1 |  | 4 | >4 | ≤0.25 - >4 |  | ≤2 | ≤2 | ≤2 - ≤2 |
| *S. sciuri* | 1 | 1 | ≤0.5 - >4 |  | ≤0.25 | 0.5 | ≤0.25 - >4 |  | ≤2 | ≤2 | ≤2 - ≤2 |
| *S. capitis* | ≤0.5 | 1 | ≤0.5 - 1 |  | ≤0.25 | 0.5 | ≤0.25 - 0.5 |  | ≤2 | ≤2 | ≤2 - ≤2 |
| *S. gallinarum* | ≤0.5 | ≤0.5 | ≤0.5 - ≤0.5 |  | 0.5 | 0.5 | ≤0.25 - 1 |  | ≤2 | ≤2 | ≤2 - ≤2 |
| *S. warneri* | ≤0.5 | ≤0.5 | ≤0.5 - ≤0.5 |  | ≤0.25 | 0.5 | ≤0.25 - >4 |  | ≤2 | ≤2 | ≤2 - ≤2 |
| *S. saprophyticus* | ≤0.5 | ≤0.5 | ≤0.5 - ≤0.5 |  | ≤0.25 | 0.5 | ≤0.25 - 0.5 |  | ≤2 | ≤2 | ≤2 - ≤2 |
| *S. arlettae* | ≤0.5 | ≤0.5 | ≤0.5 - ≤0.5 |  | >4 | >4 | >4 - >4 |  | ≤2 | ≤2 | ≤2 - ≤2 |
| *S. succinus* | ≤0.5 | ≤0.5 | ≤0.5 - ≤0.5 |  | 0.5 | 0.5 | ≤0.25 - 0.5 |  | ≤2 | ≤2 | ≤2 - ≤2 |
| *S. agnetis* | ≤0.5 | ≤0.5 | ≤0.5 - ≤0.5 |  | ≤0.25 | 0.5 | ≤0.25 - 0.5 |  | ≤2 | ≤2 | ≤2 - ≤2 |
| *S. hominis* | ≤0.5 | ≤0.5 | ≤0.5 - ≤0.5 |  | ≤0.25 | 0.5 | ≤0.25 - 0.5 |  | ≤2 | ≤2 | ≤2 - ≤2 |
| NAS | ≤0.5 | ≤0.5 | ≤0.5 - >4 |  | ≤0.25 | 0.5 | ≤0.25 - >4 |  | ≤2 | ≤2 | ≤2 - 4 |
|  |  |  |  |  |  |  |  |  |  |  |  |
| Species | LEVO | | |  | LNZ | | |  | MOX | | |
|  | MIC_50_ | MIC_90_ | Range |  | MIC_50_ | MIC_90_ | Range |  | MIC_50_ | MIC_90_ | Range |
| *S. chromogenes* | ≤0.25 | ≤0.25 | ≤0.25 - 0.5 |  | 2 | 2 | ≤1 - 4 |  | ≤0.25 | ≤0.25 | ≤0.25 - 0.5 |
| *S. simulans* | ≤0.25 | ≤0.25 | ≤0.25 - 0.5 |  | 2 | 4 | ≤1 - 4 |  | ≤0.25 | ≤0.25 | ≤0.25 - ≤0.25 |
| *S. xylosus* | 0.5 | 0.5 | ≤0.25 - 1 |  | ≤1 | 2 | ≤1 - 4 |  | ≤0.25 | ≤0.25 | ≤0.25 - ≤0.25 |
| *S. haemolyticus* | ≤0.25 | ≤0.25 | ≤0.25 - 0.5 |  | ≤1 | ≤1 | ≤1 - 2 |  | ≤0.25 | ≤0.25 | ≤0.25 - ≤0.25 |
| *S. epidermidis* | ≤0.25 | ≤0.25 | ≤0.25 - 0.5 |  | ≤1 | 2 | ≤1 - 4 |  | ≤0.25 | ≤0.25 | ≤0.25 - ≤0.25 |
| *S. cohnii* | 0.5 | 0.5 | ≤0.25 - 1 |  | 2 | 2 | ≤1 - 4 |  | ≤0.25 | ≤0.25 | ≤0.25 - ≤0.25 |
| *S. sciuri* | 0.5 | 0.5 | ≤0.25 - 1 |  | 2 | 2 | ≤1 - 2 |  | ≤0.25 | 0.5 | ≤0.25 - 0.5 |
| *S. capitis* | ≤0.25 | 0.5 | ≤0.25 - 0.5 |  | 2 | 2 | ≤1 - 2 |  | ≤0.25 | ≤0.25 | ≤0.25 - ≤0.25 |
| *S. gallinarum* | ≤0.25 | 0.5 | 0.5 - 1 |  | 2 | 4 | ≤1 - 4 |  | ≤0.25 | ≤0.25 | ≤0.25 - ≤0.25 |
| *S. warneri* | ≤0.25 | ≤0.25 | ≤0.25 - ≤0.25 |  | ≤1 | 2 | ≤1 - 2 |  | ≤0.25 | ≤0.25 | ≤0.25 - ≤0.25 |
| *S. saprophyticus* | 0.5 | 0.5 | ≤0.25 - 0.5 |  | 2 | 4 | ≤1 - 4 |  | ≤0.25 | ≤0.25 | ≤0.25 - ≤0.25 |
| *S. arlettae* | 0.5 | 0.5 | ≤0.25 - 0.5 |  | 2 | 2 | ≤1 - 2 |  | ≤0.25 | ≤0.25 | ≤0.25 - ≤0.25 |
| *S. succinus* | 0.5 | 0.5 | ≤0.25 - 0.5 |  | ≤1 | ≤1 | 2 - 4 |  | ≤0.25 | ≤0.25 | ≤0.25 - ≤0.25 |
| *S. agnetis* | ≤0.25 | ≤0.25 | ≤0.25 - ≤0.25 |  | 2 | 4 | ≤1 - 4 |  | ≤0.25 | ≤0.25 | ≤0.25 - ≤0.25 |
| *S. hominis* | ≤0.25 | ≤0.25 | ≤0.25 - ≤0.25 |  | ≤1 | ≤1 | ≤1 - ≤1 |  | ≤0.25 | ≤0.25 | ≤0.25 - ≤0.25 |
| NAS | ≤0.25 | 0.5 | ≤0.25 - 1 |  | 2 | 2 | ≤1 - 4 |  | ≤0.25 | ≤0.25 | ≤0.25 - 0.5 |

**Table S2.** **(cont.)**

| Species | NIT^1^ | | |  | OXA | | |  | PEN | | |
| --- | --- | --- | --- | --- | --- | --- | --- | --- | --- | --- | --- |
|  | MIC_50_ | MIC_90_ | Range |  | MIC_50_ | MIC_90_ | Range |  | MIC_50_ | MIC_90_ | Range |
| *S. chromogenes* | ≤32 | ≤32 | ≤32 - ≤32 |  | ≤0.25 | 0.5 | ≤0.25 - 1 |  | ≤0.06 | 1 | ≤0.06 - >8 |
| *S. simulans* | ≤32 | ≤32 | ≤32 - ≤32 |  | ≤0.25 | ≤0.25 | ≤0.25 - 1 |  | ≤0.06 | ≤0.06 | ≤0.06 - 4 |
| *S. xylosus* | ≤32 | ≤32 | ≤32 - ≤32 |  | ≤0.25 | 0.5 | ≤0.25 - 1 |  | ≤0.06 | ≤0.06 | ≤0.06 - >8 |
| *S. haemolyticus* | ≤32 | ≤32 | ≤32 - ≤32 |  | ≤0.25 | ≤0.25 | ≤0.25 - 0.5 |  | ≤0.06 | 0.25 | ≤0.06 - 2 |
| *S. epidermidis* | ≤32 | ≤32 | ≤32 - ≤32 |  | ≤0.25 | 0.5 | ≤0.25 - >4 |  | ≤0.06 | 0.5 | ≤0.06 - >8 |
| *S. cohnii* | ≤32 | ≤32 | ≤32 - ≤32 |  | 0.5 | 1 | ≤0.25 - 1 |  | ≤0.06 | 0.25 | ≤0.06 - 0.25 |
| *S. sciuri* | ≤32 | ≤32 | ≤32 - ≤32 |  | 1 | 1 | 0.5 - 2 |  | ≤0.06 | ≤0.06 | ≤0.06 - 0.25 |
| *S. capitis* | ≤32 | ≤32 | ≤32 - ≤32 |  | ≤0.25 | ≤0.25 | ≤0.25 - 0.5 |  | ≤0.06 | ≤0.06 | ≤0.06 - 0.12 |
| *S. gallinarum* | ≤32 | ≤32 | ≤32 - ≤32 |  | 0.5 | 1 | ≤0.25 - 1 |  | 0.12 | 0.12 | ≤0.06 - 0.25 |
| *S. warneri* | ≤32 | ≤32 | ≤32 - ≤32 |  | ≤0.25 | 0.5 | ≤0.25 - 0.5 |  | ≤0.06 | 0.5 | ≤0.06 - 1 |
| *S. saprophyticus* | ≤32 | ≤32 | ≤32 - ≤32 |  | 0.5 | 0.5 | ≤0.25 - 1 |  | 0.12 | 0.25 | ≤0.06 - 0.25 |
| *S. arlettae* | ≤32 | ≤32 | ≤32 - ≤32 |  | 0.5 | 1 | ≤0.25 - 1 |  | 0.25 | 0.5 | ≤0.06 - 0.5 |
| *S. succinus* | ≤32 | ≤32 | ≤32 - ≤32 |  | ≤0.25 | 0.5 | ≤0.25 - 0.5 |  | ≤0.06 | 0.12 | ≤0.06 - 0.12 |
| *S. agnetis* | ≤32 | ≤32 | ≤32 - ≤32 |  | ≤0.25 | 0.5 | ≤0.25 - 1 |  | ≤0.06 | ≤0.06 | ≤0.06 - ≤0.06 |
| *S. hominis* | ≤32 | ≤32 | ≤32 - ≤32 |  | ≤0.25 | ≤0.25 | ≤0.25 - ≤0.25 |  | ≤0.06 | ≤0.06 | ≤0.06 - 0.12 |
| NAS | ≤32 | ≤32 | ≤32 - ≤32 |  | ≤0.25 | 0.5 | ≤0.25 - >4 |  | ≤0.06 | 0.25 | ≤0.06 - >8 |
|  |  |  |  |  |  |  |  |  |  |  |  |
| Species | PNV | | |  | PIR | | |  | Q-D | | |
|  | MIC_50_ | MIC_90_ | Range |  | MIC_50_ | MIC_90_ | Range |  | MIC_50_ | MIC_90_ | Range |
| *S. chromogenes* | ≤1/2 | ≤1/2 | ≤1/2 - 4/8 |  | ≤0.5 | 1 | ≤0.5 - >4 |  | ≤0.5 | ≤0.5 | ≤0.5 - 1 |
| *S. simulans* | ≤1/2 | ≤1/2 | ≤1/2 - ≤1/2 |  | ≤0.5 | 1 | ≤0.5 - >4 |  | ≤0.5 | ≤0.5 | ≤0.5 - 4 |
| *S. xylosus* | ≤1/2 | ≤1/2 | ≤1/2 - 4/8 |  | ≤0.5 | 4 | ≤0.5 - >4 |  | 1 | 1 | ≤0.5 - 4 |
| *S. haemolyticus* | ≤1/2 | ≤1/2 | ≤1/2 - 2/4 |  | ≤0.5 | 1 | ≤0.5 - >4 |  | ≤0.5 | ≤0.5 | ≤0.5 - 2 |
| *S. epidermidis* | ≤1/2 | ≤1/2 | ≤1/2 - 2/4 |  | ≤0.5 | 4 | ≤0.5 - >4 |  | ≤0.5 | ≤0.5 | ≤0.5 - 4 |
| *S. cohnii* | ≤1/2 | ≤1/2 | ≤1/2 - ≤1/2 |  | ≤0.5 | 4 | ≤0.5 - >4 |  | 1 | 1 | ≤0.5 - 2 |
| *S. sciuri* | ≤1/2 | ≤1/2 | ≤1/2 - ≤1/2 |  | ≤0.5 | 4 | ≤0.5 - >4 |  | 1 | 2 | ≤0.5 - 2 |
| *S. capitis* | ≤1/2 | ≤1/2 | ≤1/2 - ≤1/2 |  | ≤0.5 | 1 | ≤0.5 - 4 |  | ≤0.5 | ≤0.5 | ≤0.5 - ≤0.5 |
| *S. gallinarum* | ≤1/2 | ≤1/2 | ≤1/2 - ≤1/2 |  | ≤0.5 | 1 | ≤0.5 - 1 |  | 2 | 2 | ≤0.5 - 4 |
| *S. warneri* | ≤1/2 | ≤1/2 | ≤1/2 - ≤1/2 |  | ≤0.5 | 1 | ≤0.5 - >4 |  | ≤0.5 | ≤0.5 | ≤0.5 - ≤0.5 |
| *S. saprophyticus* | ≤1/2 | ≤1/2 | ≤1/2 - ≤1/2 |  | 1 | 4 | ≤0.5 - 4 |  | ≤0.5 | 1 | ≤0.5 - 1 |
| *S. arlettae* | ≤1/2 | ≤1/2 | ≤1/2 - ≤1/2 |  | ≤0.5 | 4 | ≤0.5 - >4 |  | 1 | 1 | ≤0.5 - 1 |
| *S. succinus* | ≤1/2 | ≤1/2 | ≤1/2 - ≤1/2 |  | ≤0.5 | ≤0.5 | ≤0.5 - 1 |  | ≤0.5 | 1 | ≤0.5 - 1 |
| *S. agnetis* | ≤1/2 | ≤1/2 | ≤1/2 - ≤1/2 |  | ≤0.5 | ≤0.5 | ≤0.5 - ≤0.5 |  | ≤0.5 | ≤0.5 | ≤0.5 - ≤0.5 |
| *S. hominis* | ≤1/2 | ≤1/2 | ≤1/2 - ≤1/2 |  | ≤0.5 | 2 | ≤0.5 - 2 |  | ≤0.5 | ≤0.5 | ≤0.5 - ≤0.5 |
| NAS | ≤1/2 | ≤1/2 | ≤1/2 - 4/8 |  | ≤0.5 | 2 | ≤0.5 - >4 |  | ≤0.5 | 1 | ≤0.5 - 4 |

**Table S2.** **(cont.)**

| Species | RIF^1^ | | |  | SXT | | |  | TET | | |
| --- | --- | --- | --- | --- | --- | --- | --- | --- | --- | --- | --- |
|  | MIC_50_ | MIC_90_ | Range |  | MIC_50_ | MIC_90_ | Range |  | MIC_50_ | MIC_90_ | Range |
| *S. chromogenes* | ≤0.5 | ≤0.5 | ≤0.5 - ≤0.5 |  | ≤0.5/9.5 | ≤0.5/9.5 | ≤0.5/9.5 - 1/19 |  | ≤2 | ≤2 | ≤2 - >16 |
| *S. simulans* | ≤0.5 | ≤0.5 | ≤0.5 - ≤0.5 |  | ≤0.5/9.5 | ≤0.5/9.5 | ≤0.5/9.5 - 2/38 |  | ≤2 | >16 | ≤2 - >16 |
| *S. xylosus* | ≤0.5 | ≤0.5 | ≤0.5 - ≤0.5 |  | ≤0.5/9.5 | ≤0.5/9.5 | ≤0.5/9.5 - 1/19 |  | ≤2 | >16 | ≤2 - >16 |
| *S. haemolyticus* | ≤0.5 | ≤0.5 | ≤0.5 - 4 |  | ≤0.5/9.5 | ≤0.5/9.5 | ≤0.5/9.5 - ≤0.5/9.5 |  | ≤2 | ≤2 | ≤2 - >16 |
| *S. epidermidis* | ≤0.5 | ≤0.5 | ≤0.5 - ≤0.5 |  | ≤0.5/9.5 | ≤0.5/9.5 | ≤0.5/9.5 - >4/76 |  | ≤2 | >16 | ≤2 - >16 |
| *S. cohnii* | ≤0.5 | ≤0.5 | ≤0.5 - ≤0.5 |  | ≤0.5/9.5 | ≤0.5/9.5 | ≤0.5/9.5 - 1/19 |  | ≤2 | >16 | ≤2 - >16 |
| *S. sciuri* | ≤0.5 | ≤0.5 | ≤0.5 - ≤0.5 |  | ≤0.5/9.5 | ≤0.5/9.5 | ≤0.5/9.5 - ≤0.5/9.5 |  | ≤2 | ≤2 | ≤2 - >16 |
| *S. capitis* | ≤0.5 | ≤0.5 | ≤0.5 - ≤0.5 |  | ≤0.5/9.5 | ≤0.5/9.5 | ≤0.5/9.5 - ≤0.5/9.5 |  | ≤2 | ≤2 | ≤2 - >16 |
| *S. gallinarum* | ≤0.5 | ≤0.5 | ≤0.5 - ≤0.5 |  | ≤0.5/9.5 | ≤0.5/9.5 | ≤0.5/9.5 - ≤0.5/9.5 |  | ≤2 | ≤2 | ≤2 - >16 |
| *S. warneri* | ≤0.5 | ≤0.5 | ≤0.5 - ≤0.5 |  | ≤0.5/9.5 | ≤0.5/9.5 | ≤0.5/9.5 - ≤0.5/9.5 |  | ≤2 | ≤2 | ≤2 - >16 |
| *S. saprophyticus* | ≤0.5 | ≤0.5 | ≤0.5 - ≤0.5 |  | ≤0.5/9.5 | ≤0.5/9.5 | ≤0.5/9.5 - ≤0.5/9.5 |  | ≤2 | >16 | ≤2 - >16 |
| *S. arlettae* | ≤0.5 | ≤0.5 | ≤0.5 - ≤0.5 |  | ≤0.5/9.5 | ≤0.5/9.5 | ≤0.5/9.5 - ≤0.5/9.5 |  | ≤2 | >16 | ≤2 - >16 |
| *S. succinus* | ≤0.5 | ≤0.5 | ≤0.5 - ≤0.5 |  | ≤0.5/9.5 | ≤0.5/9.5 | ≤0.5/9.5 - ≤0.5/9.5 |  | ≤2 | ≤2 | ≤2 - ≤2 |
| *S. agnetis* | ≤0.5 | ≤0.5 | ≤0.5 - ≤0.5 |  | ≤0.5/9.5 | ≤0.5/9.5 | ≤0.5/9.5 - ≤0.5/9.5 |  | ≤2 | ≤2 | ≤2 - ≤2 |
| *S. hominis* | ≤0.5 | ≤0.5 | ≤0.5 - ≤0.5 |  | ≤0.5/9.5 | ≤0.5/9.5 | ≤0.5/9.5 - ≤0.5/9.5 |  | ≤2 | >16 | ≤2 - >16 |
| NAS | ≤0.5 | ≤0.5 | ≤0.5 - 4 |  | ≤0.5/9.5 | ≤0.5/9.5 | ≤0.5/9.5 - >4/76 |  | ≤2 | >16 | ≤2 - >16 |
|  |  |  |  |  |  |  |  |  |  |  |  |
| Species | TGC | | |  | VAN | | |  |  |  |  |
|  | MIC_50_ | MIC_90_ | Range |  | MIC_50_ | MIC_90_ | Range |  |  |  |  |
| *S. chromogenes* | 0.12 | 0.25 | ≤0.03 - 0.5 |  | 1 | 1 | ≤0.25 - 2 |  |  |  |  |
| *S. simulans* | 0.25 | 0.25 | 0.06 - 1 |  | 1 | 1 | ≤0.25 - 2 |  |  |  |  |
| *S. xylosus* | 0.25 | 0.25 | ≤0.03 - 0.5 |  | 1 | 2 | ≤0.25 - 4 |  |  |  |  |
| *S. haemolyticus* | 0.12 | 0.25 | 0.06 - 0.5 |  | 1 | 1 | ≤0.25 - 2 |  |  |  |  |
| *S. epidermidis* | 0.12 | 0.5 | 0.06 - 1 |  | 2 | 2 | 1 - 2 |  |  |  |  |
| *S. cohnii* | 0.25 | 0.25 | 0.06 - 0.5 |  | 1 | 1 | 0.5 - 2 |  |  |  |  |
| *S. sciuri* | 0.06 | 0.25 | ≤0.03 - 0.25 |  | 0.5 | 1 | ≤0.25 - 1 |  |  |  |  |
| *S. capitis* | 0.12 | 0.25 | 0.12 - 0.25 |  | 1 | 2 | 0.5 - 2 |  |  |  |  |
| *S. gallinarum* | 0.12 | 0.25 | 0.12 - 0.5 |  | 2 | 2 | 1 - 2 |  |  |  |  |
| *S. warneri* | 0.12 | 0.25 | 0.06 - 0.25 |  | 1 | 1 | 0.5 - 1 |  |  |  |  |
| *S. saprophyticus* | 0.12 | 0.5 | 0.12 - 0.5 |  | 1 | 2 | 0.5 - 2 |  |  |  |  |
| *S. arlettae* | 0.12 | 0.25 | 0.06 - 0.25 |  | 1 | 1 | 1 - 1 |  |  |  |  |
| *S. succinus* | 0.12 | 0.25 | 0.12 - 0.5 |  | 2 | 2 | 1 - 2 |  |  |  |  |
| *S. agnetis* | 0.06 | 0.12 | 0.06 - 0.25 |  | 1 | 1 | 0.5 - 1 |  |  |  |  |
| *S. hominis* | 0.12 | 0.25 | ≤0.03 - 0.25 |  | 1 | 1 | 0.5 - 1 |  |  |  |  |
| NAS | 0.12 | 0.25 | ≤0.03 - 1 |  | 1 | 2 | ≤0.25 - 4 |  |  |  |  |

^1^AMP = ampicillin; CEF = ceftiofur; CEP = cephalothin; CHL = chloramphenicol; CIP = ciprofloxacin; CLI = clindamycin; DAP = daptomycin; ERY = erythromycin, GEN = gentamicin, LEVO = levofloxacin; LNZ = linezolid; MOX = moxifloxacin; NIT = nitrofurantoin; OXA = oxacillin + 2% NaCl; PEN = penicillin; PNV = penicillin/novobiocin; PIR = pirlimycin; Q-D = quinupristin/dalfopristin; RIF = rifampin, SXT = trimethoprim/sulfamethoxazole; TET = tetracycline; TGC = tigecycline; VAN = vancomycin
