# Supplementary material for: Prevalence and Genetic Basis of Antimicrobial Resistance in Non-aureus Staphylococci Isolated from Canadian Dairy Herds
Source: Front Microbiol. 2018 Feb 16;9:256. doi: 10.3389/fmicb.2018.00256 (PMC5820348; doi:10.3389/fmicb.2018.00256)
Supplement: Supplementary file 3 [file Table3.DOCX]

Supplementary Material

**Prevalence and Genetic Basis of Antimicrobial Resistance in Non-*aureus* Staphylococci Isolated from Canadian Dairy Herds**

**Diego B. Nobrega*^1,2^, Sohail Naushad^1,2^, S. Ali Naqvi^1,2^, Larissa A. Z. Condas^1,2^, Vineet Saini^1,2,3^, John P. Kastelic^1^, Christopher Luby^2,4^, Jeroen De Buck^1,2^, and Herman W. Barkema^1,2,5^**

**^*^Correspondence:**

Dr. Diego B Nobrega

[diego.nobrega@ucalgary.ca](mailto:diego.nobrega@ucalgary.ca)

**Table S3.** Prevalence (and 95% confidence interval) of antimicrobial resistance determinants (ARDs) associated with various antimicrobial classes in different non-*aureus* staphylococci (NAS) species isolated from Canadian dairy herds.

| Antimicrobial class | ARD | NAS Species^1^ | | | | | | |
| --- | --- | --- | --- | --- | --- | --- | --- | --- |
|  |  | SAG | SAR | SAU | SCA | SCP | SCH | SCO |
| Aminoglycosides | *aac(6′)* | 0 (0-0.28) | 0 (0-0.25) | 0 (0-0.84) | 0 (0-0.17) | 0 (0-0.98) | 0 (0-0.06) | 0 (0-0.16) |
|  | *ant(3′′)* | 0 (0-0.28) | 0 (0-0.25) | 0 (0-0.84) | 0 (0-0.17) | 0 (0-0.98) | 0.03 (0-0.11) | 0.05 (0-0.24) |
|  | *ant(4′)* | 0 (0-0.28) | 0 (0-0.25) | 0 (0-0.84) | 0 (0-0.17) | 0 (0-0.98) | 0.02 (0-0.09) | 0 (0-0.16) |
|  | *ant(6)* | 0 (0-0.28) | 0 (0-0.25) | 0 (0-0.84) | 0 (0-0.17) | 0 (0-0.98) | 0.03 (0-0.11) | 0.14 (0.03-0.36) |
|  | *aph(3′)* | 0 (0-0.28) | 0 (0-0.25) | 0 (0-0.84) | 1.00 (0.83-1.00) | 0 (0-0.98) | 0 (0-0.06) | 0 (0-0.16) |
|  | *spd* | 0 (0-0.28) | 0.08 (0-0.36) | 0 (0-0.84) | 0 (0-0.17) | 0 (0-0.98) | 0 (0-0.06) | 0.05 (0-0.24) |
| Amphenicols | *fexA* | 0 (0-0.28) | 0 (0-0.25) | 0 (0-0.84) | 0 (0-0.17) | 0 (0-0.98) | 0.02 (0-0.09) | 0 (0-0.16) |
| β-Lactams | *blaZ* | 0 (0-0.28) | 0 (0-0.25) | 1.00 (0.16-1.00) | 0.20 (0.06-0.44) | 0 (0-0.98) | 0.10 (0.04-0.20) | 0.05 (0-0.24) |
|  | *mecA* | 0 (0-0.28) | 0 (0-0.25) | 0 (0-0.84) | 0 (0-0.17) | 0 (0-0.98) | 0 (0-0.06) | 0 (0-0.16) |
| Fluoroquinolones | *parC*^4^ |  |  |  |  |  |  |  |
|  | P144S | 0 (0-0.28) | 0 (0-0.25) | 0 (0-0.84) | 0 (0-0.17) | 0 (0-0.98) | 0 (0-0.06) | 0 (0-0.16) |
|  | *parE*^4^ |  |  |  |  |  |  |  |
|  | N404S | 0 (0-0.28) | 0 (0-0.25) | 0 (0-0.84) | 0 (0-0.17) | 0 (0-0.98) | 0 (0-0.06) | 0 (0-0.16) |
| Lipopeptides | *cls*^4^ |  |  |  |  |  |  |  |
|  | T33N | 0 (0-0.28) | 0 (0-0.25) | 0 (0-0.84) | 0 (0-0.17) | 0 (0-0.98) | 0 (0-0.06) | 0 (0-0.16) |
|  | *mprF*^4^ |  |  |  |  |  |  |  |
|  | G61V | 0 (0-0.28) | 0 (0-0.25) | 0 (0-0.84) | 0 (0-0.17) | 0 (0-0.98) | 0 (0-0.06) | 0 (0-0.16) |
|  | I420L | 0 (0-0.28) | 0 (0-0.25) | 0 (0-0.84) | 1.00 (0.83-1.00) | 1.00 (0.03-1.00) | 0 (0-0.06) | 0 (0-0.16) |
| MDR Efflux Pumps | DHA fam.^5^ | 0 (0-0.28) | 0.92 (0.50-0.99) | 1.00 (0.16-1.00) | 1.00 (0.83-1.00) | 1.00 (0.03-1.00) | 0 (0-0.06) | 1.00 (0.84-1.00) |
|  | *mepA* | 0 (0-0.28) | 0 (0-0.25) | 0 (0-0.84) | 0 (0-0.17) | 1.00 (0.03-1.00) | 0 (0-0.06) | 0 (0-0.16) |
|  | *norA* | 1.00 (0.72-1.00) | 1.00 (0.75-1.00) | 1.00 (0.16-1.00) | 1.00 (0.83-1.00) | 1.00 (0.03-1.00) | 1.00 (0.94-1.00) | 0.99 (0-1.00) |
|  | *norB* | 1.00 (0.72-1.00) | 0 (0-0.25) | 0 (0-0.84) | 0 (0-0.17) | 0 (0-0.98) | 0 (0-0.06) | 1.00 (0.84-1.00) |
|  | Sav1866 | 1.00 (0.72-1.00) | 1.00 (0.75-1.00) | 1.00 (0.16-1.00) | 0.99 (0-1.00) | 1.00 (0.03-1.00) | 1.00 (0.94-1.00) | 1.00 (0.84-1.00) |
| MLS^2^ | *ermA* | 0 (0-0.28) | 0 (0-0.25) | 0 (0-0.84) | 0 (0-0.17) | 0 (0-0.98) | 0 (0-0.06) | 0.05 (0-0.24) |

**Table S3.** **(cont.)**

| Antimicrobial class | ARD | NAS Species^1^ | | | | | | |
| --- | --- | --- | --- | --- | --- | --- | --- | --- |
|  |  | SAG | SAR | SAU | SCA | SCP | SCH | SCO |
| MLS^2^ | *ermC* | 0 (0-0.28) | 0 (0-0.25) | 0 (0-0.84) | 0 (0-0.17) | 0 (0-0.98) | 0 (0-0.06) | 0 (0-0.16) |
|  | *ermT* | 0 (0-0.28) | 0 (0-0.25) | 0 (0-0.84) | 0 (0-0.17) | 0 (0-0.98) | 0.03 (0-0.11) | 0 (0-0.16) |
|  | *mphC* | 0 (0-0.28) | 0.92 (0.25-1.00) | 0 (0-0.84) | 0 (0-0.17) | 0 (0-0.98) | 0 (0-0.06) | 0.14 (0.03-0.36) |
|  | *msrA* | 0 (0-0.28) | 0.92 (0.61-0.99) | 0 (0-0.84) | 0 (0-0.17) | 0 (0-0.98) | 0 (0-0.06) | 0.78 (0-1.00) |
|  | *vga* | 0 (0-0.28) | 0 (0-0.25) | 0 (0-0.84) | 0 (0-0.17) | 0 (0-0.98) | 0.03 (0-0.11) | 0 (0-0.16) |
|  | *vgbB* | 0 (0-0.28) | 0 (0-0.25) | 0 (0-0.84) | 0 (0-0.17) | 0 (0-0.98) | 0 (0-0.06) | 0 (0-0.16) |
| QAC^3^ | *qacAB* | 0 (0-0.28) | 0 (0-0.25) | 0 (0-0.84) | 0 (0-0.17) | 0 (0-0.98) | 0 (0-0.06) | 0 (0-0.16) |
| Tetracyclines | *tet38* | 1.00 (0.72-1.00) | 0 (0-0.25) | 0 (0-0.84) | 0 (0-0.17) | 0 (0-0.98) | 1.00 (0.94-1.00) | 0 (0-0.16) |
|  | *tetK* | 0 (0-0.28) | 0.15 (0.02-0.45) | 0 (0-0.84) | 0.05 (0-0.25) | 0 (0-0.98) | 0.02 (0-0.09) | 0.10 (0.01-0.30) |
|  | *tetL* | 0 (0-0.28) | 0 (0-0.25) | 0 (0-0.84) | 0.05 (0-0.25) | 0 (0-0.98) | 0.03 (0-0.11) | 0 (0-0.16) |
|  | *tetM* | 0 (0-0.28) | 0 (0-0.25) | 0 (0-0.84) | 0 (0-0.17) | 0 (0-0.98) | 0 (0-0.06) | 0 (0-0.16) |
| Sulfonamides | *folP*^4^ |  |  |  |  |  |  |  |
|  | V30I | 0.27 (0.06-0.61) | 0 (0-0.25) | 0 (0-0.84) | 0 (0-0.17) | 1.00 (0.03-1.00) | 0 (0-0.06) | 0 (0-0.16) |
|  | M37I | 0 (0-0.28) | 1.00 (0.75-1.00) | 0 (0-0.84) | 1.00 (0.83-1.00) | 0 (0-0.98) | 0 (0-0.06) | 1.00 (0.84-1.00) |
|  | I58V | 1.00 (0.72-1.00) | 0 (0-0.25) | 1.00 (0.16-1.00) | 1.00 (0.83-1.00) | 1.00 (0.03-1.00) | 1.00 (0.94-1.00) | 0 (0-0.16) |
|  | T59S | 0 (0-0.28) | 1.00 (0.75-1.00) | 0 (0-0.84) | 1.00 (0.83-1.00) | 0 (0-0.98) | 0 (0-0.06) | 1.00 (0.84-1.00) |
|  | V60L | 0 (0-0.28) | 0 (0-0.25) | 1.00 (0.16-1.00) | 1.00 (0.83-1.00) | 1.00 (0.03-1.00) | 0 (0-0.06) | 0 (0-0.16) |
|  | L64M | 0 (0-0.28) | 0 (0-0.25) | 1.00 (0.16-1.00) | 1.00 (0.83-1.00) | 1.00 (0.03-1.00) | 1.00 (0.94-1.00) | 0 (0-0.16) |
|  | I101M | 0 (0-0.28) | 1.00 (0.75-1.00) | 1.00 (0.16-1.00) | 1.00 (0.83-1.00) | 0 (0-0.98) | 0 (0-0.06) | 1.00 (0.84-1.00) |
|  | V117I | 0 (0-0.28) | 0 (0-0.25) | 0 (0-0.84) | 0 (0-0.17) | 0 (0-0.98) | 0 (0-0.06) | 0.99 (0-1.00) |
|  | V126I | 1.00 (0.72-1.00) | 0 (0-0.25) | 0 (0-0.84) | 0 (0-0.17) | 0 (0-0.98) | 1.00 (0.94-1.00) | 1.00 (0.84-1.00) |
| Aminocoumarins | *gyrB*^4^ |  |  |  |  |  |  |  |
|  | D89G | 0 (0-0.28) | 0 (0-0.25) | 0 (0-0.84) | 0 (0-0.17) | 0 (0-0.98) | 0 (0-0.06) | 1.00 (0.84-1.00) |
|  | R144I | 0 (0-0.28) | 0 (0-0.25) | 0 (0-0.84) | 0.05 (0-0.25) | 0 (0-0.98) | 0 (0-0.06) | 0 (0-0.16) |

**Table S3** **(cont.)**

| Antimicrobial class | ARD | NAS Species^1^ | | | | | | |
| --- | --- | --- | --- | --- | --- | --- | --- | --- |
|  |  | SDE | SEP | SEQ | SFL | SGA | SHA | SHO |
| Aminoglycosides | *aac(6′)* | 0 (0-0.37) | 0.04 (0-0.21) | 0 (0-0.22) | 0 (0-0.84) | 0 (0-0.19) | 0 (0-0.13) | 0 (0-0.28) |
|  | *ant(3′′)* | 0 (0-0.37) | 0 (0-0.14) | 0 (0-0.22) | 0 (0-0.84) | 0 (0-0.19) | 0 (0-0.13) | 0 (0-0.28) |
|  | *ant(4′)* | 0 (0-0.37) | 0 (0-0.14) | 0 (0-0.22) | 0 (0-0.84) | 0 (0-0.19) | 0 (0-0.13) | 0 (0-0.28) |
|  | *ant(6)* | 0 (0-0.37) | 0 (0-0.14) | 0.27 (0.08-0.55) | 0 (0-0.84) | 0 (0-0.19) | 0 (0-0.13) | 0.18 (0.02-0.52) |
|  | *aph(3′)* | 0 (0-0.37) | 0 (0-0.14) | 0 (0-0.22) | 0 (0-0.84) | 0 (0-0.19) | 0 (0-0.13) | 0 (0-0.28) |
|  | *spd* | 0 (0-0.37) | 0 (0-0.14) | 0.13 (0.02-0.40) | 0 (0-0.84) | 0 (0-0.19) | 0 (0-0.13) | 0 (0-0.28) |
| Amphenicols | *fexA* | 0 (0-0.37) | 0 (0-0.14) | 0 (0-0.22) | 0 (0-0.84) | 0 (0-0.19) | 0 (0-0.13) | 0 (0-0.28) |
| β-Lactams | *blaZ* | 0.13 (0-0.53) | 0.80 (0.17-0.99) | 0 (0-0.22) | 0 (0-0.84) | 0 (0-0.19) | 0.19 (0.06-0.38) | 0 (0-0.28) |
|  | *mecA* | 0 (0-0.37) | 0.17 (0.05-0.37) | 0 (0-0.22) | 1.00^6^ | 0 (0-0.19) | 0 (0-0.13) | 0 (0-0.28) |
| Fluoroquinolones | *parC*^4^ |  |  |  |  |  |  |  |
|  | P144S | 0.25 (0.03-0.65) | 0 (0-0.14) | 0 (0-0.22) | 0 (0-0.84) | 0 (0-0.19) | 0 (0-0.13) | 0 (0-0.28) |
|  | *parE*^4^ |  |  |  |  |  |  |  |
|  | N404S | 0 (0-0.37) | 0.04 (0-0.21) | 0 (0-0.22) | 0 (0-0.84) | 0 (0-0.19) | 0 (0-0.13) | 0 (0-0.28) |
| Lipopeptides | *cls*^4^ |  |  |  |  |  |  |  |
|  | T33N | 0 (0-0.37) | 0 (0-0.14) | 0 (0-0.22) | 0 (0-0.84) | 0 (0-0.19) | 0 (0-0.13) | 0 (0-0.28) |
|  | *mprF*^4^ |  |  |  |  |  |  |  |
|  | G61V | 0 (0-0.37) | 0 (0-0.14) | 0 (0-0.22) | 1.00 (0.16-1.00) | 0 (0-0.19) | 0 (0-0.13) | 0 (0-0.28) |
|  | I420L | 1.00 (0.63-1.00) | 1.00 (0.86-1.00) | 0 (0-0.22) | 0 (0-0.84) | 0 (0-0.19) | 0 (0-0.13) | 0 (0-0.28) |
| MDR Efflux Pumps | DHA fam.^5^ | 1.00 (0.63-1.00) | 0.96 (0.76-0.99) | 1.00 (0.78-1.00) | 0 (0-0.84) | 1.00 (0.81-1.00) | 1.00 (0.87-1.00) | 1.00 (0.72-1.00) |
|  | *mepA* | 0.99 (0-1.00) | 0 (0-0.14) | 0 (0-0.22) | 0 (0-0.84) | 0 (0-0.19) | 1.00 (0.87-1.00) | 0 (0-0.28) |
|  | *norA* | 0.99 (0-1.00) | 1.00 (0.86-1.00) | 1.00 (0.78-1.00) | 0 (0-0.84) | 1.00 (0.81-1.00) | 1.00 (0.87-1.00) | 1.00 (0.72-1.00) |
|  | *norB* | 0 (0-0.37) | 0 (0-0.14) | 1.00 (0.78-1.00) | 0 (0-0.84) | 1.00 (0.81-1.00) | 0.99 (0-1.00) | 0 (0-0.28) |
|  | Sav1866 | 1.00 (0.63-1.00) | 1.00 (0.86-1.00) | 1.00 (0.78-1.00) | 1.00 (0.16-1.00) | 1.00 (0.81-1.00) | 1.00 (0.87-1.00) | 1.00 (0.72-1.00) |
| MLS^2^ | *ermA* | 0 (0-0.37) | 0 (0-0.14) | 0 (0-0.22) | 0 (0-0.84) | 0 (0-0.19) | 0 (0-0.13) | 0 (0-0.28) |
|  | *ermC* | 0 (0-0.37) | 0.04 (0-0.21) | 0.07 (0-0.32) | 0 (0-0.84) | 0 (0-0.19) | 0 (0-0.13) | 0 (0-0.28) |
|  | *ermT* | 0 (0-0.37) | 0 (0-0.14) | 0 (0-0.22) | 0 (0-0.84) | 0 (0-0.19) | 0 (0-0.13) | 0 (0-0.28) |

**Table S3** **(cont.)**

| Antimicrobial class | ARD | NAS Species^1^ | | | | | | |
| --- | --- | --- | --- | --- | --- | --- | --- | --- |
|  |  | SDE | SEP | SEQ | SFL | SGA | SHA | SHO |
| MLS^2^ | *mphC* | 0 (0-0.37) | 0 (0-0.14) | 0.99 (0-1.00) | 1.00 (0.16-1.00) | 0 (0-0.19) | 0 (0-0.13) | 0 (0-0.28) |
|  | *msrA* | 0 (0-0.37) | 0 (0-0.14) | 0.87 (0.59-0.97) | 0 (0-0.84) | 0 (0-0.19) | 0 (0-0.13) | 0 (0-0.28) |
|  | *vga* | 0 (0-0.37) | 0.04 (0-0.21) | 0 (0-0.22) | 0 (0-0.84) | 0 (0-0.19) | 0.07 (0.01-0.24) | 0 (0-0.28) |
|  | *vgbB* | 0 (0-0.37) | 0 (0-0.14) | 0 (0-0.22) | 0 (0-0.84) | 0 (0-0.19) | 0 (0-0.13) | 0 (0-0.28) |
| QAC^3^ | *qacAB* | 0 (0-0.37) | 0.08 (0.01-0.27) | 0 (0-0.22) | 0 (0-0.84) | 0 (0-0.19) | 0.04 (0-0.19) | 0.09 (0-0.41) |
| Tetracyclines | *tet38* | 0 (0-0.37) | 0.04 (0-0.21) | 0 (0-0.22) | 0 (0-0.84) | 0 (0-0.19) | 0 (0-0.13) | 0 (0-0.28) |
|  | *tetK* | 0.13 (0-0.53) | 0.38 (0.21-0.58) | 0.07 (0-0.32) | 0 (0-0.84) | 0.11 (0.01-0.35) | 0.07 (0.01-0.24) | 0.36 (0.11-0.69) |
|  | *tetL* | 0 (0-0.37) | 0 (0-0.14) | 0 (0-0.22) | 0 (0-0.84) | 0 (0-0.19) | 0 (0-0.13) | 0 (0-0.28) |
|  | *tetM* | 0 (0-0.37) | 0 (0-0.14) | 0 (0-0.22) | 0 (0-0.84) | 0 (0-0.19) | 0 (0-0.13) | 0 (0-0.28) |
| Sulfonamides | *folP*^4^ |  |  |  |  |  |  |  |
|  | V30I | 0 (0-0.37) | 0 (0-0.14) | 1.00 (0.78-1.00) | 0 (0-0.84) | 0 (0-0.19) | 0 (0-0.13) | 0 (0-0.28) |
|  | M37I | 1.00 (0.63-1.00) | 0.96 (0.76-0.99) | 0 (0-0.22) | 1.00 (0.16-1.00) | All | 0.89 (0.71-0.96) | 1.00 (0.72-1.00) |
|  | I58V | 0 (0-0.37) | 1.00 (0.86-1.00) | 0 (0-0.22) | 1.00 (0.16-1.00) | 0 (0-0.19) | 0 (0-0.13) | 1.00 (0.72-1.00) |
|  | T59S | 1.00 (0.63-1.00) | 0.96 (0.76-0.99) | 1.00 (0.78-1.00) | 1.00 (0.16-1.00) | 0.06 (0-0.27) | 1.00 (0.87-1.00) | 1.00 (0.72-1.00) |
|  | V60L | 0 (0-0.37) | 0.99 (0-1.00) | 0 (0-0.22) | 1.00 (0.16-1.00) | 0 (0-0.19) | 0 (0-0.13) | 1.00 (0.72-1.00) |
|  | L64M | 0 (0-0.37) | 1.00 (0.86-1.00) | 0 (0-0.22) | 0 (0-0.84) | 0.94 (0.69-0.99) | 0 (0-0.13) | 1.00 (0.72-1.00) |
|  | I101M | 1.00 (0.63-1.00) | 1.00 (0.86-1.00) | 1.00 (0.78-1.00) | 1.00 (0.16-1.00) | 1.00 (0.81-1.00) | 1.00 (0.87-1.00) | 1.00 (0.72-1.00) |
|  | V117I | 0 (0-0.37) | 0 (0-0.14) | 0 (0-0.22) | 0 (0-0.84) | 0 (0-0.19) | 0 (0-0.13) | 0 (0-0.28) |
|  | V126I | 0 (0-0.37) | 0 (0-0.14) | 0 (0-0.22) | 0 (0-0.84) | 0 (0-0.19) | 0 (0-0.13) | 0 (0-0.28) |
| Aminocoumarins | *gyrB*^4^ |  |  |  |  |  |  |  |
|  | D89G | 0 (0-0.37) | 0 (0-0.14) | 1.00 (0.78-1.00) | 0 (0-0.84) | 1.00 (0.81-1.00) | 0.04 (0-0.19) | 0 (0-0.28) |
|  | R144I | 0 (0-0.37) | 0 (0-0.14) | 0 (0-0.22) | 0 (0-0.84) | 0 (0-0.19) | 0 (0-0.13) | 0 (0-0.28) |

**Table S3** **(cont.)**

| Antimicrobial class | ARD | NAS Species^1^ | | | | | | |
| --- | --- | --- | --- | --- | --- | --- | --- | --- |
|  |  | SHY | SKL | SNE | SPA | SSA | SSC | SSI |
| Aminoglycosides | *aac(6′)* | 0 (0-0.71) | 0 (0-0.98) | 0 (0-0.84) | 0 (0-0.46) | 0 (0-0.22) | 0 (0-0.13) | 0 (0-0.09) |
|  | *ant(3′′)* | 0.33 (0.01-0.91) | 0 (0-0.98) | 0 (0-0.84) | 0 (0-0.46) | 0 (0-0.22) | 0 (0-0.13) | 0 (0-0.09) |
|  | *ant(4′)* | 0 (0-0.71) | 0 (0-0.98) | 0 (0-0.84) | 0 (0-0.46) | 0 (0-0.22) | 0.04 (0-0.20) | 0 (0-0.09) |
|  | *ant(6)* | 0 (0-0.71) | 0 (0-0.98) | 0 (0-0.84) | 0.33 (0.04-0.78) | 0.13 (0.02-0.40) | 0 (0-0.13) | 0 (0-0.09) |
|  | *aph(3′)* | 0 (0-0.71) | 0 (0-0.98) | 0 (0-0.84) | 0 (0-0.46) | 0 (0-0.22) | 0 (0-0.13) | 0 (0-0.09) |
|  | *spd* | 0 (0-0.71) | 0 (0-0.98) | 0 (0-0.84) | 0 (0-0.46) | 0 (0-0.22) | 0 (0-0.13) | 0 (0-0.09) |
| Amphenicols | *fexA* | 0 (0-0.71) | 0 (0-0.98) | 0 (0-0.84) | 0 (0-0.46) | 0 (0-0.22) | 0 (0-0.13) | 0.05 (0.01-0.18) |
| β-Lactams | *blaZ* | 0 (0-0.71) | 0 (0-0.98) | 0 (0-0.84) | 0.17 (0-0.64) | 0 (0-0.22) | 0 (0-0.13) | 0 (0-0.09) |
|  | *mecA* | 0 (0-0.71) | 0 (0-0.98) | 0 (0-0.84) | 0 (0-0.46) | 0 (0-0.22) | 1.00^6^ | 0 (0-0.09) |
| Fluoroquinolones | *parC*^4^ |  |  |  |  |  |  |  |
|  | P144S | 0 (0-0.71) | 0 (0-0.98) | 0 (0-0.84) | 0 (0-0.46) | 0 (0-0.22) | 0 (0-0.13) | 0 (0-0.09) |
|  | *parE*^4^ |  |  |  |  |  |  |  |
|  | N404S | 0 (0-0.71) | 0 (0-0.98) | 0 (0-0.84) | 0 (0-0.46) | 0 (0-0.22) | 0 (0-0.13) | 0 (0-0.09) |
| Lipopeptides | *cls*^4^ |  |  |  |  |  |  |  |
|  | T33N | 0 (0-0.71) | 0 (0-0.98) | 0 (0-0.84) | 0 (0-0.46) | 0 (0-0.22) | 0 (0-0.13) | 0.11 (0.03-0.25) |
|  | *mprF*^4^ |  |  |  |  |  |  |  |
|  | G61V | 0 (0-0.71) | 0 (0-0.98) | 0 (0-0.84) | 0 (0-0.46) | 0 (0-0.22) | 1.00 (0.87-1.00) | 0 (0-0.09) |
|  | I420L | 0 (0-0.71) | 0 (0-0.98) | 0 (0-0.84) | 0 (0-0.46) | 0 (0-0.22) | 0 (0-0.13) | 1.00 (0.91-1.00) |
| MDR Efflux Pumps | DHA fam.^5^ | 0 (0-0.71) | 1.00 (0.03-1.00) | 1.00 (0.16-1.00) | 1.00 (0.54-1.00) | 1.00 (0.78-1.00) | 0 (0-0.13) | 0.05 (0.01-0.18) |
|  | *mepA* | 0 (0-0.71) | 0 (0-0.98) | 0 (0-0.84) | 1.00 (0.54-1.00) | 0.99 (0-1.00) | 0 (0-0.13) | 0 (0-0.09) |
|  | *norA* | 1.00 (0.29-1.00) | 0 (0-0.98) | 1.00 (0.16-1.00) | 1.00 (0.54-1.00) | 1.00 (0.78-1.00) | 0 (0-0.13) | 1.00 (0.91-1.00) |
|  | *norB* | 1.00 (0.29-1.00) | 0 (0-0.98) | 1.00 (0.16-1.00) | 0 (0-0.46) | 1.00 (0.78-1.00) | 0 (0-0.13) | 0 (0-0.09) |
|  | Sav1866 | 1.00 (0.29-1.00) | 1.00 (0.03-1.00) | 1.00 (0.16-1.00) | 1.00 (0.54-1.00) | 1.00 (0.78-1.00) | 1.00 (0.87-1.00) | 1.00 (0.91-1.00) |
| MLS^2^ | *ermA* | 0 (0-0.71) | 0 (0-0.98) | 0 (0-0.84) | 0 (0-0.46) | 0 (0-0.22) | 0 (0-0.13) | 0 (0-0.09) |
|  | *ermC* | 0 (0-0.71) | 0 (0-0.98) | 0 (0-0.84) | 0 (0-0.46) | 0 (0-0.22) | 0 (0-0.13) | 0 (0-0.09) |
|  | *ermT* | 0 (0-0.71) | 0 (0-0.98) | 0 (0-0.84) | 0 (0-0.46) | 0 (0-0.22) | 0 (0-0.13) | 0 (0-0.09) |

**Table S3** **(cont.)**

| Antimicrobial class | ARD | NAS Species^1^ | | | | | | |
| --- | --- | --- | --- | --- | --- | --- | --- | --- |
|  |  | SHY | SKL | SNE | SPA | SSA | SSC | SSI |
| MLS^2^ | *mphC* | 0 (0-0.71) | 0 (0-0.98) | 0.50 (0.01-0.99) | 0 (0-0.46) | 0 (0-0.22) | 1.00 (0.87-1.00) | 0 (0-0.09) |
|  | *msrA* | 0 (0-0.71) | 0 (0-0.98) | 0 (0-0.84) | 0 (0-0.46) | 0 (0-0.22) | 0 (0-0.13) | 0 (0-0.09) |
|  | *vga* | 0 (0-0.71) | 0 (0-0.98) | 0 (0-0.84) | 0 (0-0.46) | 0 (0-0.22) | 0 (0-0.13) | 0.03 (0-0.14) |
|  | *vgbB* | 0 (0-0.71) | 0 (0-0.98) | 0 (0-0.84) | 0 (0-0.46) | 0 (0-0.22) | 0 (0-0.13) | 0 (0-0.09) |
| QAC^3^ | *qacAB* | 0 (0-0.71) | 0 (0-0.98) | 0 (0-0.84) | 0 (0-0.46) | 0 (0-0.22) | 0 (0-0.13) | 0 (0-0.09) |
| Tetracyclines | *tet38* | 1.00 (0.29-1.00) | 0 (0-0.98) | 0 (0-0.84) | 0 (0-0.46) | 0 (0-0.22) | 0 (0-0.13) | 0 (0-0.09) |
|  | *tetK* | 0.33 (0.01-0.91) | 0 (0-0.98) | 0.50 (0.01-0.99) | 0.50 (0.12-0.88) | 0.33 (0.12-0.62) | 0 (0-0.13) | 0.03 (0-0.14) |
|  | *tetL* | 0 (0-0.71) | 0 (0-0.98) | 0 (0-0.84) | 0 (0-0.46) | 0 (0-0.22) | 0.08 (0.01-0.25) | 0.03 (0-0.14) |
|  | *tetM* | 0 (0-0.71) | 0 (0-0.98) | 0 (0-0.84) | 0 (0-0.46) | 0 (0-0.22) | 0 (0-0.13) | 0.03 (0-0.14) |
| Sulfonamides | *folP*^4^ |  |  |  |  |  |  |  |
|  | V30I | 0 (0-0.71) | 0 (0-0.98) | 1.00 (0.16-1.00) | 0 (0-0.46) | 0 (0-0.22) | 0 (0-0.13) | 0 (0-0.09) |
|  | M37I | 0 (0-0.71) | 1.00 (0.03-1.00) | 1.00 (0.16-1.00) | 0.83 (0.36-1.00) | 0 (0-0.22) | 1.00 (0.87-1.00) | 0 (0-0.09) |
|  | I58V | 1.00 (0.29-1.00) | 1.00 (0.03-1.00) | 0 (0-0.84) | 0 (0-0.46) | 0 (0-0.22) | 1.00 (0.87-1.00) | 1.00 (0.91-1.00) |
|  | T59S | 0 (0-0.71) | 1.00 (0.03-1.00) | 1.00 (0.16-1.00) | 0 (0-0.46) | 0 (0-0.22) | 1.00 (0.87-1.00) | 0 (0-0.09) |
|  | V60L | 0 (0-0.71) | 0 (0-0.98) | 0 (0-0.84) | 1.00 (0.54-1.00) | 0 (0-0.22) | 1.00 (0.87-1.00) | 1.00 (0.91-1.00) |
|  | L64M | 0 (0-0.71) | 0 (0-0.98) | 0 (0-0.84) | 0 (0-0.46) | 0 (0-0.22) | 0 (0-0.13) | 0 (0-0.09) |
|  | I101M | 0 (0-0.71) | 1.00 (0.03-1.00) | 1.00 (0.16-1.00) | 1.00 (0.54-1.00) | 1.00 (0.78-1.00) | 1.00 (0.87-1.00) | 1.00 (0.91-1.00) |
|  | V117I | 0 (0-0.71) | 1.00 (0.03-1.00) | 0 (0-0.84) | 0 (0-0.46) | 1.00 (0.78-1.00) | 0 (0-0.13) | 0 (0-0.09) |
|  | V126I | 1.00 (0.29-1.00) | 0 (0-0.98) | 0 (0-0.84) | 0 (0-0.46) | 0 (0-0.22) | 0 (0-0.13) | 0.03 (0-0.14) |
| Aminocoumarins | *gyrB*^4^ |  |  |  |  |  |  |  |
|  | D89G | 0 (0-0.71) | 1.00 (0.03-1.00) | 1.00 (0.16-1.00) | 0 (0-0.46) | 1.00 (0.78-1.00) | 0 (0-0.13) | 0 (0-0.09) |
|  | R144I | 0 (0-0.71) | 1.00 (0.03-1.00) | 0 (0-0.84) | 0 (0-0.46) | 0 (0-0.22) | 0 (0-0.13) | 0 (0-0.09) |

**Table S3** **(cont.)**

| Antimicrobial class | ARD | NAS Species^1^ | | | | |
| --- | --- | --- | --- | --- | --- | --- |
|  |  | SSU | SVI | SWA | SXY | NAS |
| Aminoglycosides | *aac(6′)* | 0 (0-0.22) | 0 (0-0.46) | 0 (0-0.20) | 0 (0-0.16) | 0 (0-0.01) |
|  | *ant(3′′)* | 0 (0-0.22) | 0 (0-0.46) | 0 (0-0.20) | 0 (0-0.16) | 0 (0-0.05) |
|  | *ant(4′)* | 0 (0-0.22) | 0 (0-0.46) | 0 (0-0.20) | 0 (0-0.16) | 0 (0-0.02) |
|  | *ant(6)* | 0 (0-0.22) | 0.17 (0-0.64) | 0 (0-0.20) | 0 (0-0.16) | 0.01 (0-0.04) |
|  | *aph(3′)* | 0 (0-0.22) | 0 (0-0.46) | 0.18 (0.04-0.43) | 0 (0-0.16) | 0.01 (0-0.03) |
|  | *spd* | 0 (0-0.22) | 0 (0-0.46) | 0 (0-0.20) | 0 (0-0.16) | 0 (0-0.02) |
| Amphenicols | *fexA* | 0 (0-0.22) | 0 (0-0.46) | 0 (0-0.20) | 0 (0-0.16) | 0.01 (0-0.02) |
| β-Lactams | *blaZ* | 0 (0-0.22) | 0 (0-0.46) | 0.06 (0-0.29) | 0 (0-0.16) | 0.06 (0.03-0.11) |
|  | *mecA* | 0 (0-0.22) | 0 (0-0.46) | 0 (0-0.20) | 0 (0-0.16) | 0.01 (0-0.02)^7^ |
| Fluoroquinolones | *parC*^4^ |  |  |  |  |  |
|  | P144S | 0 (0-0.22) | 0 (0-0.46) | 0 (0-0.20) | 0 (0-0.16) | 0 (0-0.01) |
|  | *parE*^4^ |  |  |  |  |  |
|  | N404S | 0 (0-0.22) | 0 (0-0.46) | 0 (0-0.20) | 0 (0-0.16) | 0 (0-0.01) |
| Lipopeptides | *cls*^4^ |  |  |  |  |  |
|  | T33N | 0 (0-0.22) | 0 (0-0.46) | 0 (0-0.20) | 0 (0-0.16) | 0 (0-0.09) |
|  | *mprF*^4^ |  |  |  |  |  |
|  | G61V | 0 (0-0.22) | 0 (0-0.46) | 0 (0-0.20) | 0 (0-0.16) | 0.04 (0.02-0.06) |
|  | I420L | 0 (0-0.22) | 0 (0-0.46) | 1.00 (0.80-1.00) | 0 (0-0.16) | 0.18 (0.12-0.25) |
| MDR Efflux Pumps | DHA fam.^5^ | 1.00 (0.78-1.00) | 0 (0-0.46) | 1.00 (0.80-1.00) | 1.00 (0.84-1.00) | 0.44 (0.36-0.52) |
|  | *mepA* | 0 (0-0.22) | 0 (0-0.46) | 1.00 (0.80-1.00) | 0 (0-0.16) | 0.12 (0.08-0.17) |
|  | *norA* | 1.00 (0.78-1.00) | 0 (0-0.46) | 1.00 (0.80-1.00) | 1.00 (0.84-1.00) | 0.96 (0.93-0.98) |
|  | *norB* | 1.00 (0.78-1.00) | 0 (0-0.46) | 0 (0-0.20) | 1.00 (0.84-1.00) | 0.29 (0.21-0.37) |
|  | Sav1866 | 1.00 (0.78-1.00) | 1.00 (0.54-1.00) | 1.00 (0.80-1.00) | 0.99 (0-1.00) | 1.00 (0.91-1.00) |
| MLS^2^ | *ermA* | 0 (0-0.22) | 0 (0-0.46) | 0 (0-0.20) | 0 (0-0.16) | 0 (0-0.01) |
|  | *ermC* | 0 (0-0.22) | 0 (0-0.46) | 0 (0-0.20) | 0 (0-0.16) | 0 (0-0.01) |
|  | *ermT* | 0 (0-0.22) | 0 (0-0.46) | 0 (0-0.20) | 0 (0-0.16) | 0.01 (0-0.03) |

**Table S3** **(cont.)**

| Antimicrobial class | ARD | NAS Species^1^ | | | | |
| --- | --- | --- | --- | --- | --- | --- |
|  |  | SSU | SVI | SWA | SXY | NAS |
| MLS^2^ | *mphC* | 0 (0-0.22) | 1.00 (0.54-1.00) | 0 (0-0.20) | 0.33 (0.17-0.55) | 0.10 (0.07-0.15) |
|  | *msrA* | 0 (0-0.22) | 0 (0-0.46) | 0 (0-0.20) | 0.14 (0.03-0.36) | 0.05 (0.03-0.09) |
|  | *vga* | 0 (0-0.22) | 0 (0-0.46) | 0 (0-0.20) | 0 (0-0.16) | 0.01 (0-0.05) |
|  | *vgbB* | 0 (0-0.22) | 0 (0-0.46) | 0 (0-0.20) | 0.05 (0-0.24) | 0 (0-0.01) |
| QAC^3^ | *qacAB* | 0 (0-0.22) | 0 (0-0.46) | 0 (0-0.20) | 0 (0-0.16) | 0.01 (0-0.02) |
| Tetracyclines | *tet38* | 0 (0-0.22) | 0 (0-0.46) | 0 (0-0.20) | 0 (0-0.16) | 0.30 (0.19-0.41) |
|  | *tetK* | 0 (0-0.22) | 0 (0-0.46) | 0 (0-0.20) | 0.19 (0.05-0.42) | 0.04 (0.02-0.08) |
|  | *tetL* | 0 (0-0.22) | 0 (0-0.46) | 0 (0-0.20) | 0 (0-0.16) | 0 (0-0.04) |
|  | *tetM* | 0 (0-0.22) | 0 (0-0.46) | 0 (0-0.20) | 0 (0-0.16) | 0 (0-0.02) |
| Sulfonamides | *folP*^4^ |  |  |  |  |  |
|  | V30I | 0 (0-0.22) | 0 (0-0.46) | 0 (0-0.20) | 0.10 (0.01-0.30) | 0.01 (0-0.05) |
|  | M37I | 0 (0-0.22) | 1.00 (0.54-1.00) | 0.45 (0.12-0.83) | 1.00 (0.84-1.00) | 0.42 (0.35-0.49) |
|  | I58V | 0 (0-0.22) | 1.00 (0.54-1.00) | 1.00 (0.80-1.00) | 1.00 (0.84-1.00) | 0.79 (0.74-0.83) |
|  | T59S | 1.00 (0.78-1.00) | 1.00 (0.54-1.00) | 0 (0-0.20) | 1.00 (0.84-1.00) | 0.42 (0.35-0.50) |
|  | V60L | 0 (0-0.22) | 1.00 (0.54-1.00) | 1.00 (0.80-1.00) | 0 (0-0.16) | 0.23 (0.17-0.30) |
|  | L64M | 0.99 (0-1.00) | 0 (0-0.46) | 0 (0-0.20) | 0 (0-0.16) | 0.45 (0.36-0.54) |
|  | I101M | 1.00 (0.78-1.00) | 1.00 (0.54-1.00) | 1.00 (0.80-1.00) | 1.00 (0.84-1.00) | 0.72 (0.59-0.82) |
|  | V117I | 0 (0-0.22) | 0 (0-0.46) | 0 (0-0.20) | 1.00 (0.84-1.00) | 0.08 (0.04-0.15) |
|  | V126I | 0 (0-0.22) | 0 (0-0.46) | 0 (0-0.20) | 0 (0-0.16) | 0.39 (0.30-0.49) |
| Aminocoumarins | *gyrB*^4^ |  |  |  |  |  |
|  | D89G | 1.00 (0.78-1.00) | 0 (0-0.46) | 0 (0-0.20) | 1.00 (0.84-1.00) | 0.16 (0.10-0.24) |
|  | R144I | 0 (0-0.22) | 0 (0-0.46) | 0 (0-0.20) | 0 (0-0.16) | 0 (0-0.01) |

^1^SAG = *S. agnetis*; SAR = *S. arlettae*; SAU = *S. auricularis*; SCA = *S. capitis*; SCP = *S. caprae*; SCH = *S. chromogenes*; SCO = *S. cohnii*; SDE = *S. devriesei*; SEP = *S. epidermidis*; SEQ = *S. equorum*; SFL = *S. fleuretti*; SGA = *S. gallinarum*; SHA = *S. haemolyticus*; SHO = *S. hominis*; SHY = *S. hyicus*; SKL = *S. kloosii*; SNE = *S. nepalensis*; SPA = *S. pasteuri*; SSA = *S. saprophyticus*; SSC = *S. sciuri*; SSI = *S. simulans*; SSU = *S. succinus*; SVI = *S. vitulinus*; SWA = *S. warneri*; SXY = *S. xylosus*; All = all NAS grouped

^2^Macrolides, lincosamides and streptogramins

^3^Quaternary ammonium compounds

^4^Specific substitution in the deduced amino acid sequence

^5^DHA family of MFS transporters

^6^*mecA* variants (*mecA sf*, *mecA1*)

^7^Prevalence estimation ignoring *mecA* variants
